# Mobile Health Self-management Support for Spinal Cord Injury: Systematic Literature Review

**DOI:** 10.2196/42679

**Published:** 2023-04-26

**Authors:** Renaldo M Bernard, Vanessa Seijas, Micheal Davis, Anel Volkova, Nicola Diviani, Janina Lüscher, Carla Sabariego

**Affiliations:** 1 Swiss Paraplegic Research Nottwil Switzerland; 2 Faculty of Health Sciences and Medicine University of Lucerne Lucerne Switzerland; 3 Center for Rehabilitation in Global Health Systems World Health Organization Collaborating Center University of Lucerne Lucerne Switzerland

**Keywords:** mobile phone, mobile health, mHealth, eHealth, telemedicine, telehealth, spinal cord injury, self-management, internet-based intervention, World Wide Web, systematic review

## Abstract

**Background:**

Self-management plays a critical role in maintaining and improving the health of persons with spinal cord injury (SCI). Despite their potential, existing mobile health (mHealth) self-management support (SMS) tools for SCI have not been comprehensively described in terms of their characteristics and approaches. It is important to have an overview of these tools to know how best to select, further develop, and improve them.

**Objective:**

The objective of this systematic literature review was to identify mHealth SMS tools for SCI and summarize their characteristics and approaches to offering SMS.

**Methods:**

A systematic review of the literature published between January 2010 and March 2022 was conducted across 8 bibliographic databases. The data synthesis was guided by the self-management task taxonomy by Corbin and Strauss, the self-management skill taxonomy by Lorig and Holman, and the Practical Reviews in Self-Management Support taxonomy. The PRISMA (Preferred Reporting Items for Systematic Reviews and Meta-Analyses) standards guided the reporting.

**Results:**

A total of 24 publications reporting on 19 mHealth SMS tools for SCI were included. These tools were introduced from 2015 onward and used various mHealth technologies and multimedia formats to provide SMS using 9 methods identified by the Practical Reviews in Self-Management Support taxonomy (eg, social support and lifestyle advice and support). The identified tools focused on common SCI self-management areas (eg, bowel, bladder, and pain management) and overlooked areas such as sexual dysfunction problems and environmental problems, including barriers in the built environment. Most tools (12/19, 63%) unexpectedly supported a single self-management task instead of all 3 tasks (ie, medical, role, and emotional management), and emotional management tasks had very little support. All self-management skills (eg, problem-solving, decision-making, and action planning) had coverage, but a single tool addressed resource use. The identified mHealth SMS tools were similar in terms of number, introduction period, geographical distribution, and technical sophistication compared with SMS tools for other chronic conditions.

**Conclusions:**

This systematic literature review provides one of the first descriptions of mHealth SMS tools for SCI in terms of their characteristics and approaches to offering SMS. This study’s findings highlight a need for increased coverage of key SMS for SCI components; adopting comparable usability, user experience, and accessibility evaluation methods; and related research to provide more detailed reporting. Future research should consider other data sources such as app stores and technology-centric bibliographic databases to complement this compilation by identifying other possibly overlooked mHealth SMS tools. A consideration of this study’s findings is expected to support the selection, development, and improvement of mHealth SMS tools for SCI.

## Introduction

### Background

Spinal cord injury (SCI) is a complex chronic health condition that carries a high health, economic, and social burden for those affected and their families. SCI can be traumatic or nontraumatic in nature and is characterized by the loss or impairment of motor, sensory, or autonomic functions below the level of the injury [1]. Frequent health complications include pressure injury, urinary tract infections, bowel dysfunction, mental health conditions, pulmonary complications, pain, and sexual dysfunction [2,3]. The limitations in functioning caused by SCIs are largely dependent on the neurological level and severity of the injury, associated comorbidities and complications, the age of onset, available health and social care resources, and the presence of barriers or facilitators in the person’s environment [1]. Similarly, wider participation in society is also made difficult without a concerted effort to pursue further education or sustainable employment, sufficient financial support, and the alleviation of comorbidities [4].

Self-management plays a critical role in maintaining and improving the health of persons with SCI [5]. It is widely understood as the ability of an individual to manage the symptoms, treatment, biopsychosocial consequences, and lifestyle changes inherent to living with a chronic health condition [6]. Corbin and Strauss [7] introduced 3 tasks, namely, medical, role, and emotional management, that describe how people with chronic health conditions manage their health. Lorig and Holman [8] described 6 key skills that support the execution of these tasks: problem-solving, decision-making, resource use, forming patient-provider partnerships, action planning, and self-tailoring. Pearce et al [9] argued that self-management is nonetheless not the sole responsibility of persons affected by chronic health conditions and proposed the Practical Reviews in Self-Management Support (PRISMS) taxonomy to highlight 14 self-management support (SMS) activities such as the provision of social support and equipment. SMS is often provided in the form of traditional institutional and paper-based options [6]. However, technology-based SMS options help overcome traditional barriers of distance, time, and high economic costs and are increasingly becoming available for SCI [10,11].

The use of technology-based SMS for chronic conditions has expanded with the widespread adoption of mobile health (mHealth) technology [10]. Compared with early desktop computer–based technologies, mHealth provides more person-centered, available, accessible, and scalable tools [12]. It introduces the use of mobile and wireless information and communications technologies, including geospatial services, movement, light and proximity sensors, and Bluetooth technology, bundled into mobile devices, apps, and wearable technologies, among other similar products, to support meeting health needs [13]. In the context of SMS, this could involve using a mobile device to receive visual, auditory, and tactile-based reminders to perform a health behavior (eg, taking medication), self-monitor health status (eg, recording vital signs), learn from web-based informational resources, and secure social support from online peer groups [9,14]. mHealth is well positioned to benefit from the high adoption rates among persons with SCI. Over 87% of participants with traumatic SCI in a 2018 study indicated that they were mobile internet users, which represented a 35% increase from 2012 [15] and a 12% higher rate than the global mobile internet subscription rate in 2019 [16]. An increase in the global user base has also been attributed to the recent pandemic [17], which is also expected to have a similar impact among persons with SCI in the last 2 years.

Nonetheless, to the best of our knowledge, the available mHealth SMS for SCI has not been comprehensively compiled. Reviews on the closest related topics have focused on accessing telerehabilitation [10], telehealth care [18], and telecounseling [19-21] outside clinical settings but have not adequately considered SMS and, with the exception of one study [10], mHealth. The latest review was also completed in early 2016, which does not account for the expected rapid increase in the development of mHealth options over the last 6 years. Therefore, it is important to have an overview of available mHealth SMS options for SCI.

### Objectives

The objective of this systematic literature review was to identify and summarize the mHealth SMS tools developed for SCI. It aimed to describe their volume, features, evidence base, and reporting and recommend future directions for the development, evaluation, and reporting of these tools. Articulating data on effectiveness, gaps in coverage, usability shortcomings, and impact is expected to help patients and clinicians with selecting tools and support researchers and developers in optimizing existing tools or deciding and planning the development of new ones.

## Methods

### Overview

A systematic review was conducted to identify and summarize the mHealth SMS tools for SCI. The PRISMA (Preferred Reporting Items for Systematic Reviews and Meta-Analyses) guidelines [22] and the extension for searching [23] were used to guide reporting (Multimedia Appendix 1). The inclusion of an assessment of methodological quality for other types of observational studies overlooked by the study protocol [24] was the single protocol deviation observed.

### Search Strategy

MEDLINE, Academic Search Premier, LISTA, Business Source Premier, Scopus, CINAHL Complete, PsycINFO, and Web of Science Core Collection were searched using keywords for SCI and mHealth (Multimedia Appendix 2). The reference lists of included articles were also hand searched.

### Eligibility Criteria

Publications were eligible for inclusion if they described an mHealth [13] SMS tool [6] for SCI. Eligible mHealth SMS tools were optimized for access from mobile devices to help accommodate the accessibility needs of people with SCI and were intended for use outside a clinical setting or not dependent on assistance from others to obtain benefits. Publications including primary research studies, books, and gray literature (eg, conference proceedings, theses, government documents, and professional publications) made available in the English language between January 2010 and March 2022 were considered. Gray literature such as commentaries and letters to the editor that were unlikely to discuss mHealth SMS tools for SCI in sufficient detail were not considered.

### Eligibility Assessment

In total, 3 researchers (AV, MD, and RMB), including a health scientist, psychologist, and health technologist, were involved in screening. They attended a training workshop to help ensure consistency in screening using the web-based service Rayyan (Rayyan Systems Inc) [25] without its artificial intelligence–based features. The screeners completed a training set of 100 publications. Conflicting screening decisions (ie, *include*, *maybe*, or *exclude*) were discussed to clarify any misunderstandings. A total of 2 screeners were then randomly assigned a screening set of titles and abstracts. A third screener afterward performed a second screening of 29% (29/100) of the publications. In total, 2 screeners (AV and RMB) conducted eligibility checks on the full texts. Screening was independently conducted to reduce the risk of reviewer bias [26], and conflicting screening decisions were resolved collaboratively.

### Risk-of-Bias Assessment

The same researchers who conducted the screening (RMB and AV), along with a rehabilitation physician (VS), independently evaluated the risk of bias for the included studies based on recommendations from Ma et al [27] and according to the assessment strategy shown in Textbox 1. Disagreements in evaluations were resolved collaboratively.

**Strategy for risk-of-bias assessment.**

**Experimental studies**

- Revised version of the Cochrane Collaboration tool for assessing risk of bias in randomized trials [28]

**Mixed methods studies**

- Mixed Methods Appraisal Tool for systematic mixed studies reviews [29]

**Other observational studies**

- Joanna Briggs Institute Checklist for Analytical Cross-Sectional Studies [30]

**Qualitative studies**

- Joanna Briggs Institute Checklist for Qualitative Research [31]

**Quasi-experimental studies**

- Joanna Briggs Institute Checklist for Quasi-Experimental Studies [32]

### Data Extraction and Synthesis

MD and RMB completed the data extraction. These researchers attended a training workshop to help ensure consistency and reliability using a web-based data extraction form. This form was discussed and modified for increased clarity. One researcher extracted data from the included publications, another reviewed and verified the extracted data, and discrepancies were resolved collaboratively. The extracted data were collated and summarized by 2 researchers (RMB and AV) using a descriptive synthesis. The analysis of frequencies, except for publication characteristics, only considered unique data extracted from publications focusing on the same mHealth option. The synthesis of evaluative information considered usability [33] and user experience [34]. Data extraction and synthesis were also guided by frameworks for self-management tasks [7] and skills [8], as detailed in Textbox 2, and support activities [9]. Aspects of SMS for SCI that were targeted by the included mHealth tools [35] were described using emergent themes.

**Self-management task and skill frameworks.**

**Self-management tasks [7]**

- Medical management
  Making health-related appointments, following treatment plans, tracking symptoms, and taking medication as directed
- Role management
  Organizing and coordinating the various everyday roles and responsibilities related to work, family, community, and self-care and adapting these roles as needed
- Emotional management
  Regulating and coping with emotions resulting from living with a condition in a healthy and effective manner

**Self-management skills [8]**

- Problem-solving
  Identifying problems and finding, implementing, and evaluating solutions
- Decision-making
  Weighing options and choosing the best course of action in response to changes in their condition
- Resource use
  Finding and effectively using resources
- Forming patient-provider partnerships
  Learning from and partnering with health care professionals to understand the patterns experienced with a condition, make informed decisions, and discuss related issues
- Action planning
  Developing a realistic action plan that can be confidently used to achieve a set goal
- Self-tailoring
  Developing and implementing personalized self-management strategies as needed

## Results

### Overview

**Figure 1:**
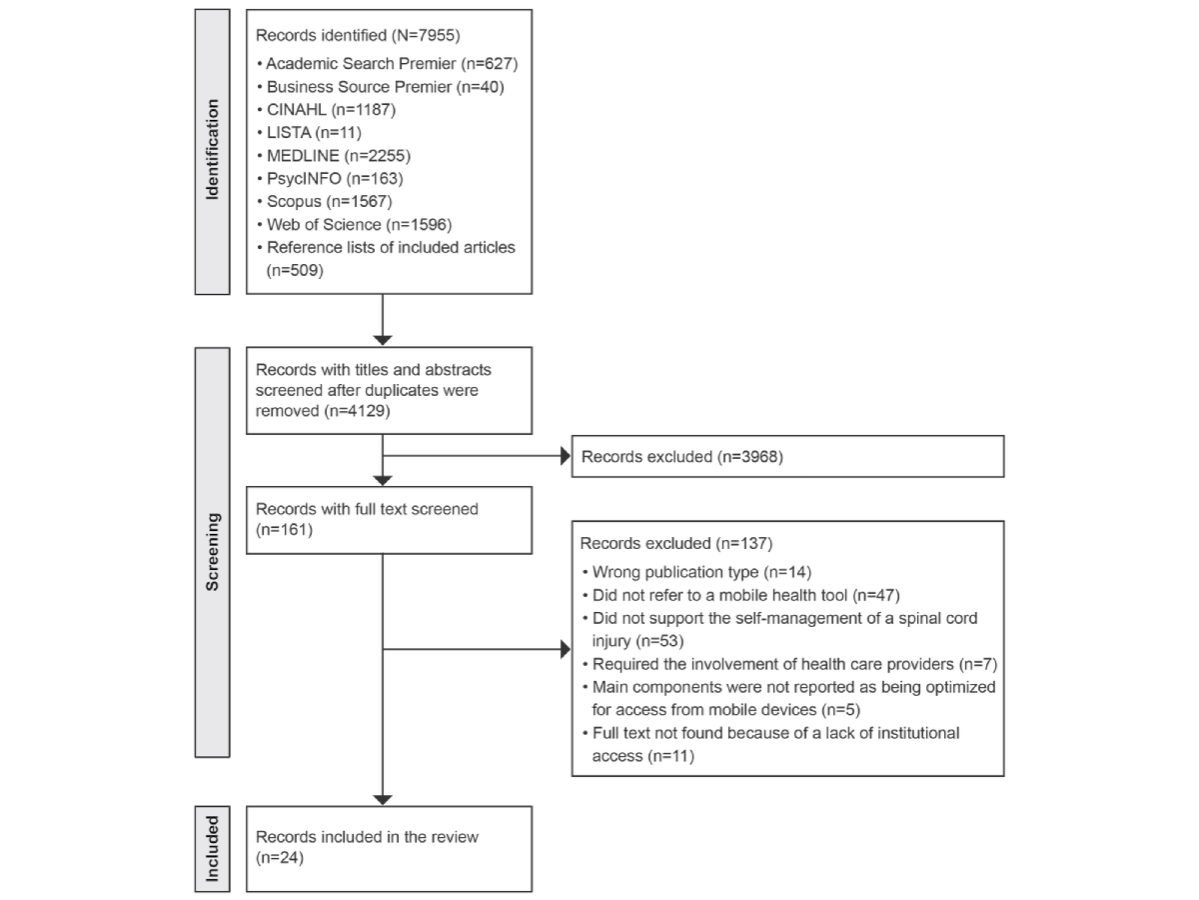
PRISMA (Preferred Reporting Items for Systematic Reviews and Meta-Analyses) flowchart of the review search, selection, and inclusion process.

A total of 24 publications [36-59] were included, and Figure 1 details the methodological process.

### Characteristics of the Included Publications

The 24 included publications comprised 20 (83%) studies [36,37,40,42-57,59], 3 (12%) reports [38,39,58], and 1 (4%) protocol paper [41] (Table S1 Multimedia Appendix 3). The included publications primarily aimed to describe and develop mHealth tools (10/24, 42%) [38,39,43,45,46,48,54,56-58], evaluate implementation factors (9/24, 38%) [37,40,45,46,49,50,54,55,57], evaluate usability and user experience (7/24, 29%) [45-48,52-54] and effectiveness (6/24, 25%) [36,41,42,44,51,54], and describe stakeholder perspectives (1/24, 4%). The included publications were published between 2015 and 2022, and most (18/24, 75%) were published from 2018 onward [41-57,59]. The research teams were mainly based in North America (15/24, 62%) [38,40-42,44-47,50,52,53,55,56,58,59], followed by Europe (6/24, 25%) [36,37,48,49,54,57], Asia (2/24, 8%) [43,51], and Europe and Asia (1/24, 4%) [39].

Of the 20 included studies, 7 (35%) were quasi-experimental [37,42,43,50,51,53,57], 5 (25%) were mixed methods [46-48,54], 4 (20%) were qualitative [49,52,56,59], 2 (10%) were observational [45,55], and 2 (10%) were experimental [36,44]. The risk of bias was deemed low in just over half (11/20, 55%) of the included studies [44,45,47,49,51,52,55-57,59] (Multimedia Appendix 4 [36-59]). No studies were excluded based on the risk assessment. Study participants experienced various limitations in body functions, including musculoskeletal and movement-related functions [37,39,43], sensory functions and pain [36,47,51,59], urination and defecation [40,45,47,50,58,59], skin [38,45-50,52,53,59], sleep [54], and functions that help manage the psychological and social demands of daily life [44,45,47,54,59]. The sample sizes of the 20 included studies ranged from 4 to 75 participants where reported [36,37,40,42-57,59]. The age range of the study participants was 18 to 81 years, and most study participants were male (232/396, 59%) where reported (19/20, 95%) [36,37,40,42,44-57,59].

### Characteristics of the Underlying mHealth Technology

A total of 19 mHealth tools were identified (Table 1 and Table S2 Multimedia Appendix 3). In total, 4% (1/24) of the publications focused on 2 tools [37], 12% (3/24) focused on 1 tool [41,47,50], and 2 sets of 2 focused on 1 tool equally (2/24, 8%) [40,48,49,58]. In total, 4 tools were unnamed (4/24, 17%) [38,40,43,48,49]. The included publications documented mHealth tools mainly at their testing stage (10/19, 53%) [36,40,43-45,47,50-52,54,55,58], followed by the developmental (6/19, 32%) [38,39,41,46,48,56,59], proof-of-concept (3/19, 16%) [37,42,49], proposal (1/19, 5%) [57], and launch (1/19, 5%) [53] stages.

The design and development of the included mHealth tools followed phases largely characteristic of user-centered design as the process was iterative and sought to understand users, the relevant tasks they needed to perform, and the environment of use (10/19, 53%) [36,39,40,44,46,47,50-52,54,57,59]. Participatory design, in which stakeholders are encouraged to make substantial contributions to design decisions, was used to a lesser extent (3/19, 16%) [45,48,49,56]. A total of 32% (6/19) of the publications [37,38,42,43,53,55] did not describe the design process adopted.

The primary technologies used were apps (16/19, 84%) [37-39,41-55,57,59], mobile-optimized websites (2/19, 11%) [40,56,58], and a glove (ie, wearable; 1/19, 5%) [36]. These technologies were mainly connected to mobile phones (10/19, 53%) [39,42-44,46,48,49,51,53,54,57], followed by tablets (7/19, 37%) [37,39,41,45,47,50,52,54,55], unspecified mobile devices (4/19, 21%) [36,38,40,56,58], pressure mats (2/19, 11%) [46,53], smartwatches (ie, wearable; 2/19, 11%) [42,55], smart garments (ie, wearable; 1/19, 5%) [39], and Raspberry Pi (1/19, 5%) [46].

The Android mobile operating system was the most frequently chosen (8/19, 42%) [39,41-44,48,50,53,57], closely followed by iOS (7/19, 37%) [37,38,45-47,52,53]. A total of 11% (2/19) of the tools used both operating systems [41,47,50,53], 11% (2/19) were operating system–agnostic [40,56,58], and 21% (4/19) did not report this information [36,51,54,55]. Further requirements regarding the device and operating system version and full language and country availability were largely vague or absent and could not be extracted.

When reported, devices required a display (18/19, 95%) [37-48,50-57,59], internet connectivity (12/19, 63%) [38,40,43-46,48,51,53-56], audio (6/19, 32%) [36,39,45,48,55,57], camera (5/19, 26%) [37,43-45,48], Bluetooth (5/19, 26%) [36,39,42,46,53], reminder features (5/19, 26%) [38,43,44,48,54], accelerometer sensor (4/19, 21%) [38,42,43,57], notification features (4/19, 21%) [38,39,42,46], messaging (3/19, 16%) [44,45,48], and cloud storage (1/19, 5%) [38]. Table 2 summarizes each requirement of the included mHealth tools. Multimedia Appendix 5 [36-59] organizes each requirement by self-management tasks, skills, and support components and tasks.

**Table 1:** Number of mobile health (mHealth) tools introduced per year (n=19).

Year of introduction	mHealth tools, n (%)
2015	3 (16)
2016	2 (11)
2017	0 (0)
2018	0 (0)
2019	7 (37)
2020	3 (16)
2021	3 (16)
2022	1 (5)

**Table 2:** Device requirements of the included mobile health tools (n=19).

Device requirement and citation	Description of use	Frequency, n (%)
Display [37-59]	Used for presenting the mobile device’s user interface in visual and tactile form	18 (95)
Internet connectivity [38,40,43-46,48,49,51,53-56,58]	Used for accessing web-based information, having voice and video calls, and sending and storing information via the web	12 (63)
Audio [36,39,45,48,49,55,57]	Used for listening to multimedia content with sound, creating audio messages, and having voice calls	6 (32)
Bluetooth [36,39,42,46,53]	Used for exchanging data over short distances between mobile devices and paired technologies	5 (26)
Camera [37,43-45,48,49]	Used for having video calls and capturing still images	5 (26)
Reminders [38,43,44,48,49,54]	Used for alerting users to participate in a planned activity	5 (26)
Accelerometer [38,42,43,57]	Embedded in a smartphone or wearable (eg, smartwatch) and used for motion sensing	4 (21)
Notifications [38,39,42,46]	Used for informing users of available mobile technology information updates via audio, visual, and tactile indicators	4 (21)
Messaging [44,45,48,49]	Used for multimedia communication via the internet	3 (16)
Cloud storage [38]	Used for data storage	1 (5)

### Characteristics of Approaches Providing SMS for SCI

The mHealth tools supported the completion of all self-management tasks (Table 3). Emotional management had little support (3/19, 16%) [44,47,54,59] compared with medical (14/19, 74%) [36-39,41,43,44,46-50,53-56,58,59] and role (12/19, 63%) [38,40-42,45,47-52,54,56-59] management tasks. Most mHealth tools supported 1 self-management task (12/19, 63%) [36,37,39,42,43,45,46,51-53,55,57], followed by 26% (5/19) supporting 2 self-management tasks [38,40,44,48,49,56,58] and 11% (2/19) supporting 3 self-management tasks [41,47,50,54,59].

The mHealth tools supported the practice of all self-management skills (Table 4). The top 4 self-management skills were supported more than the average number of times. These 4 represented 84% (31/37) of the total number of times that self-management skills were supported. Most mHealth tools (7/19, 37%) supported 1 self-management skill [41,44,47-51,56,57,59], followed by 32% (6/19) supporting 2 self-management tasks [38,40,43,45,54,55,58] and 3 self-management tasks [36,37,39,42,46,52,53].

The mHealth tools incorporated 64% (9/14) of the PRISMS support components (Table 5). The top 4 components were incorporated more than the average number of times. These 4 represented 74% (35/47) of the total number of times that the components were incorporated. Most mHealth tools (8/19, 42%) incorporated 1 support component [36,37,39,42,46,52,53,55], followed by 21% (4/19) incorporating 4 support components [40,44,51,54,58], 16% (3/19) incorporating 3 support components [38,45,48,49], and 11% (2/19) incorporating 2 support components [43,57] and 5 support components [41,47,50,56,59]. These 10 components largely focused on supporting pressure injury prevention, physical activity promotion, and bladder management (Table 3). The lowest focus was placed on spasticity management, autonomic dysreflexia management, sleep management, and shoulder posture monitoring (Table 6).

The adopted self-management approaches were individualized only or combined with a group-based approach. Individualized approaches included multimedia educational content (eg, audio, text, images, and video), real-time behavioral visualizations or illustrations, textual or haptic feedback, personalized physical movement plans, games, 2-way messaging with health care professionals, content requiring active end-user engagement (eg, diary), and progress-tracking features (16/19, 84%) [36-39,41-53,55,57,59]. Combined approaches included forums and progress-tracking leaderboards (3/19, 16%) [40,54,56,58].

The included mHealth tools were intended mostly for use in nonclinical (ie, home and community environment) settings (17/19, 89%) [37-39,42-46,48,51-58], and only 11% (2/19) were also intended for use in clinical settings [36,41,47,50,59]. The adopted approaches largely relied on research (14/19, 74%) [36,42,44-48,51,52,54,55,58], followed by theory (5/19, 26%) [38,39,41,43,56] and expertise (1/19, 5%) [57]. Approaches targeting therapeutic exercise for legs [43] and shoulder posture monitoring [39] solely relied on theory. The provision of training and practice for everyday activities that targeted therapeutic exercise for the hands was the only approach that was without an app [36].

**Table 3:** Characteristics of approaches providing self-management support for spinal cord injury (SCI; n=19).

mHealth tool name, citation, and country availability	Self-management focus areas	Relevant self-management tasks	Relevant self-management skills	Relevant self-management support components
AW-Shift^a^ [53], United States	Pressure injury management	Medical management	Decision-making	Monitoring of the condition with feedback
Ball Strike, Pop Flux [37], Italy	Therapeutic exercise for hands, legs, or trunk	Medical management	Action planning	Training and rehearsal for practical self-management activities
CMAP^c^ [46], United States	Pressure injury management	Medical management	Problem-solving	Monitoring of the condition with feedback
Fisiofriend [57], Italy	Physical activity promotion	Role management	Maintaining patient-provider partnership, action planning, and self-tailoring	Training and rehearsal for practical self-management activities and monitoring of the condition with feedback
iMHere^d^ [44], United States	Bladder management, pressure injury management, and psychosocial support	Medical and emotional management	Maintaining patient-provider partnership, self-tailoring, and decision-making	Practical support with adherence (medication or behavioral), information about the condition or its management, provision of easy access to advice or support when needed, and monitoring of the condition with feedback
MMT^e^ [36], Turkey	Therapeutic exercise for hands, legs, or trunk	Medical management	Problem-solving	Training and rehearsal for everyday activities
M2M^f^ [55], United States	Physical activity promotion	Medical management	Self-tailoring and problem-solving	Information about the condition or its management
NR [38], United States	Pressure injury management	Medical and role management	Decision-making and resource use	Practical support with adherence (medication or behavioral), information about the condition or its management, and monitoring of the condition with feedback
NR [40,58], United States	Bladder management	Role and medical management	Maintaining patient-provider partnership and problem-solving	DPractical support with adherence (medication or behavioral), information about the condition or its management, provision of easy access to advice or support when needed, and social support
NR [43], Thailand	Therapeutic exercise for hands, legs, or trunk	Medical management	Self-tailoring and decision-making	Practical support with adherence (medication or behavioral) and training and rehearsal for practical self-management activities
NR [48,49], Switzerland	Pressure injury management	Medical and role management	Maintaining patient-provider partnership, self-tailoring, and problem-solving	Practical support with adherence (medication or behavioral), information about the condition or its management, and provision of easy access to advice or support when needed
PHOENIX^h^ [45], United States	Pressure injury, bladder, and bowel management	Role management	Action planning and problem-solving	Information about the condition or its management, lifestyle advice and support, and social support
PHIRE^i^ [42], United States	Physical activity promotion	Role management	Self-tailoring	Monitoring of the condition with feedback
PUT^j^ [52], Canada	Pressure injury management	Role management	Problem-solving	Information about the condition or its management
Punsook [51], Thailand	Bladder and pain management	Role management	Maintaining patient-provider partnership, action planning, and problem-solving	Practical support with adherence (medication or behavioral), information about the condition or its management, monitoring of the condition with feedback, and provision of easy access to advice or support when needed
SCI Health Storylines [41,47,50,59], Canada	Bladder management, bowel management, pressure injury management, spasticity management, autonomic dysreflexia management, physical activity promotion, pain management, psychosocial support, medicating and dieting, sensation of pain, handling stress and other psychological demands, and looking after one’s health	Medical, role, and emotional management	Action planning, decision-making, and self-tailoring	Practical support with adherence (medication or behavioral), information about the condition or its management, training and rehearsal for practical self-management activities, monitoring of the condition with feedback, and training and rehearsal for psychological strategies
WHEELS^k^ [54], the Netherlands	Physical activity promotion, psychosocial support, sleep management, and medicating and dieting	Medical, role, and emotional management	Problem-solving and action planning	Practical support with adherence (medication or behavioral), information about the condition or its management, social support, and training and rehearsal for practical self-management activities
WOWii^l^ [56], United States	Physical activity promotion	Medical and role management	Problem-solving, action planning, and decision-making	Practical support with adherence (medication or behavioral), information about the condition or its management, social support, lifestyle advice and support, and training and rehearsal for practical self-management activities

^a^mHealth: mobile health.

^b^AW-Shift: Assisted Weight Shift.

^c^CMAP: Comprehensive Mobile Assessment of Pressure.

^d^iMHere: Interactive Mobile Health and Rehabilitation.

^e^MMT: Mobile Music Touch.

^f^M2M: Movement-to-Music.

^g^NR: not reported.

^h^PHOENIX: Peer-Supported Health Outreach, Education, and Information Exchange.

^i^PHIRE: Personal Health Informatics and Rehabilitation Engineering.

^j^PUT: Pressure Ulcer Target.

^k^WHEELS: Wheelchair Exercise and Lifestyle Study.

^l^WOWii: Workout on Wheels internet intervention.

**Table 4:** Supported self-management skills (n=19).

Self-management skills	Frequency, n (%)
Problem-solving [36,40,45, 46, 48, 49, 51, 52, 54-56, 58]	10 (53)
Decision-making [38,39,41, 43, 44, 50, 53, 56, 59]	7 (37)
Self-tailoring [42-44,47-49, 55, 57]	7 (37)
Action planning [37,41,45,47,50, 51, 54, 56, 57, 59]	7 (37)
Maintaining patient-provider partnership [40,44,48,49,51, 57, 58]	5 (26)
Resource use [38]	1 (5)

**Table 5:** Incorporated self-management support components (n=19).

Self-management support components	Frequency, n (%)
Information about the condition, its management, or both [38,40,41,44,45,47-49,51,52,54-56,58,59]	11 (58)
Practical support with adherence (medication or behavioral) [38-41,43,44,47-49,51,54,56,58,59]	10 (53)
Monitoring of the condition with feedback [38,41,42,44,46,47,51,53,57,59]	8 (42)
Training or rehearsal for practical self-management activities [37,41,43,47,50,54,56,57,59]	6 (32)
Provision of easy access to advice or support when needed [40,44,48,49,51,58]	4 (21)
Social support [40,45,54,56,58]	4 (21)
Lifestyle advice and support [45,56]	2 (11)
Training or rehearsal for everyday activities [36]	1 (5)
Training or rehearsal for psychological strategies [47,59]	1 (5)

**Table 6:** Targeted self-management focus areas (n=19).

Self-management focus areas	Frequency, n (%)
Pressure injury management [38,41,44-50,52,53,59]	8 (42)
Physical activity promotion [41,42,50,54-57]	6 (32)
Bladder management [40,41,44,45,47,50,51,58,59]	5 (26)
Psychosocial support [44,47,54,59]	3 (16)
Therapeutic exercise for hands, legs, or trunk [36,37,43]	3 (16)
Bowel management [41,45,47,50,59]	2 (11)
Pain management [47,50,51,59]	2 (11)
Medicating and dieting [47,54,59]	2 (11)
Spasticity management [41,47]	1 (5)
Autonomic dysreflexia management [41]	1 (5)
Sleep management [54]	1 (5)
Shoulder posture monitoring [39]	1 (5)

### Evaluation of mHealth SMS for SCI

**Figure 2:**
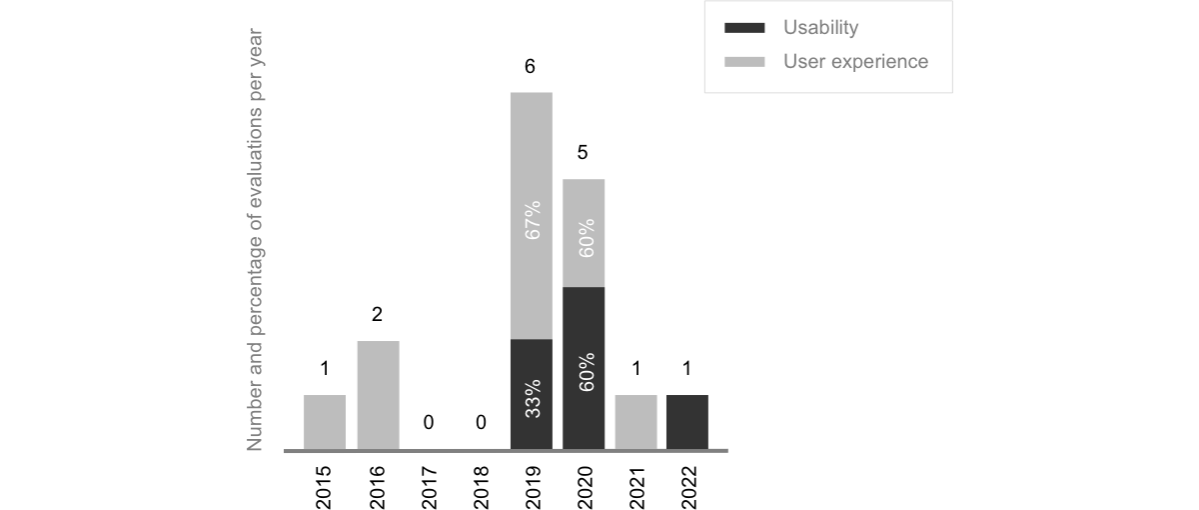
Temporal distribution of mobile health tool evaluation.

The included studies reported a significant change in trunk control [37], urinary tract infections [44], hand sensory functions [36], self-management for neurogenic bladder dysfunction [40], and bowel management confidence [50] (Table 1). No significant changes in urinary tract leakage and infections or pain [51] and psychosocial-related outcomes [44] were observed. None of the included studies published before 2019 (6/20, 30%) had the primary aim of evaluating usability or user experience. The included studies that conducted these evaluations increased 4-fold during the last 4 years (15/18, 83%) [45-48,51,54,56] compared with the previous period (3/18, 17%) [37,39,40] (Figure 2). When evaluated by 58% (14/24) of the included publications [37,40,43,45-49,51,52,54,56,57,59], interviews, focus groups, surveys, and field studies were used. A widely adopted instrument (eg, the System Usability Scale; 4/14, 29%) [46-48,54] was seldom used for evaluating usability or user experience (Table 3). All evaluations relied on empirical methods involving participants with SCI. The results from the usability evaluations were largely *very good* (6/12, 50%) [37,40,45-48,51], followed by those indicating *good* (3/12, 25%) [46-48] and *poor* (1/12, 8%) [54] usability. The user experience evaluations were mostly *good* (3/4, 75%) [43,46,57] and *very good* (1/4, 25%) [51]. Other evaluations of usability [56,59] and user experience [49,52] generated 12 change requests from study participants regarding design, content, and functionality. No accessibility evaluations were reported.

## Discussion

### Principal Findings and Comparison With Prior Work

The 24 included publications introduced 19 mHealth SMS tools for SCI since 2015 using various mHealth technologies and multimedia formats. The findings support the notion that the adoption of mHealth SMS tools for SCI is a growing area of interest [10]. The findings are similar to those of reviews of a comparable period identifying 23 heart failure– [60]; 21 cardiovascular disease– [61]; 23 HIV-, AIDS-, or HIV and AIDS– [62]; and 17 Parkinson disease–related [63] mHealth SMS tools that were introduced from 2012 onward. The geographical distribution of the tools in the included publications is also similar to that in these reviews, with the large majority of tools being introduced in North America and Europe except in the case of HIV or AIDS [62], where no tools introduced in Europe were reported.

### mHealth Technologies Underlying SMS for SCI

A review of mHealth SMS tools for heart failure [60] reported that 48% of the identified tools benefited from a participatory design approach compared with 16% (3/19) in this review. However, as evidenced in the study by Allin et al [64], adopting participatory design for the development of mHealth SMS tools for SCI is instrumental in highlighting accessibility, design, and information quality concerns and developing potential solutions in response. Nonetheless, research also highlights that a participatory design approach does not guarantee sustained engagement [64]. The remaining reviews did not report the approach adopted for designing the identified tools, which is similar to 32% (6/19) of the tools identified in this review. Unlike comparable reviews [61,62], our review found a nearly equal operating system share between Android and iOS despite the former being the most used by a large margin [65]. Unlike comparable reviews [61,63] except for that by Mehraeen et al [62], widely used wearable devices (eg, smartwatches) were not identified in this review despite their demonstrated potential for health self-management. This likely results partially from the difficulty to accurately measure physical activity in persons with SCI using wearables [66]. The identified device requirements are also common features found in many mobile devices, which allowed for the easy adoption of these tools and which were also present in and similarly used by the tools identified in comparable reviews.

### Approaches to SMS for SCI

The lack of support for all 3 self-management tasks (ie, medical, role, and emotional) was unexpected as SMS tools should aim to include content that addresses all of them [8]. The lack of support for emotional management was unexpectedly pronounced given that managing the psychological demands of SCI and other chronic conditions is a core task for those affected [67]. Compared with the 10 most common problems reported by persons with SCI [68,69], the included mHealth tools largely addressed similar problems but prioritized them differently. For example, pressure injury was ranked much lower by people with SCI than the high level of coverage this complication had in the included mHealth tools. Moreover, the included mHealth tools did not address environmental problems such as barriers in the built environment and sexual dysfunction problems. Compared with non–mHealth-based self-management interventions identified in a recent scoping review [70], the identified mHealth tools reflected a very similar level of focus on SCI symptoms. Except for sexual functions, the identified mHealth tools considered many additional symptoms and health self-management options in comparison. The interventions and mHealth tools identified in this review similarly ascertained the provision of *information about the condition and/or its management* as being the most common PRISMS support component offered. However, the findings from the recent scoping review and this review differ in their coverage of the remaining PRISMS components and self-management skills. This is likely partially due to mHealth being more suited for offering *practical support with adherence*, *monitoring of the condition with feedback*, and *provision of easy access to advice or support when needed*, for example, compared with alternative methods (eg, paper- and desktop computer–based options). The recent scoping review [70] also found that most self-management interventions following an SCI were individualized or combined with a group-based approach. The identified mHealth tools used more sophisticated but comparable formats with alternative self-management interventions [70].

### Evaluation of mHealth SMS Tools for SCI

mHealth tools require a high level of usability to ensure that they can be easily used over time without expending unwarranted effort. Comparable reviews have reported a lower percentage of usability evaluations for hypertension (2/21, 10%) [71], diabetes (14/31, 45%) [72], and heart failure (9/18, 50%) [73] than that reported by the included studies. Although the usability of the included tools was generally ranked positively, the failure to use standardized measurement instruments makes it difficult to ascertain what exactly was measured and compare with findings from similar studies. Comparable reviews have reported a slightly higher adoption of standardized instruments for usability evaluations for diabetes (6/31, 19%) [72] and heart failure (4/18, 22%) [73]. Comparable studies investigating user experience of self-management tools were few [74,75], similarly revealed positive results [74], did not adopt widely used assessment instruments [74], and benefited from qualitative methods to gain insights into improvements [74,75].

### Implications for Future Practice and Research

More effort is needed to develop mHealth SMS tools for SCI with consideration for incorporating all self-management tasks and undersupported self-management skills and support components. New approaches that can bridge the observed fragmentation of SMS provided by mHealth tools for SCI should be pursued. For example, mHealth SMS tools for chronic health conditions share several common features, and a reference architecture could be of benefit for the efficient and cost-effective development of mHealth SMS tools for SCI, other chronic health conditions, or a combination of these. These technologies are shaped by their underlying technical frameworks as much as by their features. Decisions regarding the design, development, and implementation of mHealth tools need to be reported in detail and investigated to inform future decision-making regarding mHealth tools. Usability and user experience evaluations should use commonly adopted instruments, including the System Usability Scale [76]; the Usefulness, Satisfaction, and Ease of Use Questionnaire [77]; and the Post-Study System Usability Questionnaire [78], to enhance the validity of evaluations and comparability of findings. Furthermore, empirical methods such as usability testing with users should be complemented by other methods [79], including expert inspections and automated evaluations, to improve the validity of these evaluations. Considering and reporting the supported level of functioning by an mHealth tool is essential given the considerable accessibility needs of people with SCI (eg, difficulties associated with sensory and motor impairments). Similar reviews should include more technology-centric databases, for example, the one from the Institute of Electrical and Electronics Engineers, in their search strategy. A systematic search of the most used app stores can complement this review’s findings by identifying and evaluating SMS apps for SCI that are available to the public.

### Limitations

The included publications were unlikely to account for all available mHealth SMS tools for SCI. Furthermore, one of the identified apps was retired from the Apple App Store (ie, Assisted Weight Shift) [53], another was retired from the Google Play Store (ie, Pop Flux) [37], and a single app was available from both digital distribution platforms (eg, Interactive Mobile Health and Rehabilitation) [44]. Nonetheless, this systematic literature review is necessary to comprehensively account for these tools. The mHealth tools were also insufficiently described by the included studies, and this prevented a deeper evaluation. For example, despite notable differences in the cost and features of mobile devices using the Android and iOS operating systems, it was difficult to understand how the operating system was chosen without a rationale being provided, especially when their adoption rates were almost the same. Information about the intervention, such as its name; details about primary and secondary users, including lesion type and injury etiology; the design process followed; and minimum hardware and software requirements, was vaguely reported or absent and could have provided valuable insight. For example, it might have indicated a fuller coverage of self-management tasks. This inadequate reporting might also reflect publication restrictions regarding word limits and alternative focus topics where authors instead strategically prioritize other details. Despite these shortcomings in reporting, the included studies still provided more relevant details than tools identified via other means, such as app store descriptions. The publication year restriction could have excluded otherwise eligible mHealth tools, but the findings from this study and the latest review on a related topic [10] strongly suggest that very few or no tools would have been missed as a result. Only considering mobile-optimized web-based services for inclusion likely reduced the number of web-based mHealth tools included, but it is an essential feature given the accessibility needs of people with SCIs. Usability and user experience evaluations were limited as they relied on empirical evaluations, which typically focus on testing select system tasks with users instead of all possible tasks. However, the focus is often on essential tasks, and the practice reduces costs such as time, money, and effort to conduct the evaluation [79].

### Conclusions

This systematic literature review provides one of the first overviews of mHealth SMS tools for SCI and represents one of the first steps in a wider research agenda aiming to comprehensively account for these tools. This review identified 19 mHealth tools reported across the 24 included publications and an increasing development trend. A synthesis of these findings highlighted the need for mHealth to support key underserviced SMS components for SCI, more standardized or commonly used evaluation methods for usability and user experience, and more detailed reporting that includes key technical details and decisions that shape the mHealth tool. Future research is encouraged to consider other sources for the identification of mHealth SMS tools for SCI, such as app stores and more technology-centric bibliographic databases, to complement this compilation.

## Appendix

Multimedia Appendix 1 PRISMA (Preferred Reporting Items for Systematic Reviews and Meta-Analyses) checklist.

Multimedia Appendix 2 Search concepts and terms and search strategies for the queried bibliographic databases.

Multimedia Appendix 3 Characteristics of the included publications (N=24) and included mobile health (mHealth) tools (n=19).

Multimedia Appendix 4 Risk-of-bias assessment of the included studies.

Multimedia Appendix 5 Device requirements by self-management area, skills, and support methods.

## Abbreviations

mHealth: mobile health
PRISMA: Preferred Reporting Items for Systematic Reviews and Meta-Analyses
PRISMS: Practical Reviews in Self-Management Support
SCI: spinal cord injury
SMS: self-management support
