# Supplementary material for: Mobile Health Self-management Support for Spinal Cord Injury: Systematic Literature Review
Source: JMIR Mhealth Uhealth. 2023 Apr 26;11:e42679. doi: 10.2196/42679 (PMC10173031; doi:10.2196/42679)
Supplement: Multimedia Appendix 2 [file mhealth_v11i1e42679_app2.docx]

## Multimedia Appendix 2

**Search concepts and terms, and search strategies for each bibliographic database queried**

**Table 1.** Summary of search concepts and terms

| **Concepts** | **Core search terms** |
| --- | --- |
| spinal cord injuries | spinal cord injury, paraplegic, tetraplegic, quadriplegic |
| mhealth | app, artificial intelligence, augmented reality, digital, electronic communication, informatics, information and communications, information technology, Internet, machine learning, medical informatics, mhealth, mixed reality, mobile application, mobile device, mobile healthcare, mobile phone, monitoring device, monitoring sensor, neural network, online, remote consultation, self-help device, smart device, smart glasses, smartphone, tablet, telecommunication, telemedicine, telemonitoring, teletherapy, virtual, wearable, wearable electronic devices, wireless technology |

**Table 2.** Academic Search Premier search strategy

| **#** | **Query** | **Results** |
| --- | --- | --- |
| S56 | S55 (Limiters - Published Date: 20100101-20220331; Language: English) | 627 |
| S55 | S53 AND S54 | 1,302 |
| S54 | S6 OR S7 OR S8 OR S9 OR S10 OR S11 OR S12 OR S13 OR S14 OR S15 OR S16 OR S17 OR S18 OR S19 OR S20 OR S21 OR S22 OR S23 OR S24 OR S25 OR S26 OR S27 OR S28 OR S29 OR S30 OR S31 OR S32 OR S33 OR S34 OR S35 OR S36 OR S37 OR S38 OR S39 OR S40 OR S41 OR S42 OR S43 OR S44 OR S45 OR S46 OR S47 OR S48 OR S49 OR S50 OR S51 OR S52 | 1,550,418 |
| S53 | S1 OR S2 OR S3 OR S4 OR S5 | 28,270 |
| S52 | DE "SMARTPHONES" OR DE "ATRIX (Smartphone)" OR DE "BLACKBERRY (Smartphone)" OR DE "BLACKBERRY Bold (Smartphone)" OR DE "BLACKBERRY Curve (Smartphone)" OR DE "BLACKBERRY Pearl (Smartphone)" OR DE "BLACKBERRY Storm (Smartphone)" OR DE "DROID (Smartphone)" OR DE "FIRE (Smartphone)" OR DE "G1 (Smartphone)" OR DE "HTC One (Smartphone)" OR DE "IPHONE (Smartphone)" OR DE "NEXUS One (Smartphone)" OR DE "NOKIA smartphones" OR DE "PALM Pre (Smartphone)" OR DE "SAMSUNG Galaxy Nexus (Smartphone)" OR DE "SAMSUNG Galaxy Note" OR DE "SAMSUNG Galaxy S" | 22,684 |
| S51 | DE "INFORMATION & communication technologies" OR DE "ELECTRONIC services" OR DE "INFORMATION & communication technologies for development" OR DE "TELEPRESENCE" | 11,104 |
| S50 | DE "DIGITAL health" OR DE "INDIVIDUALIZED medicine" OR DE "MOBILE health" OR DE "TELEREHABILITATION" OR DE "WEARABLE technology" | 18,667 |
| S49 | DE "MOBILE apps" OR DE "ENTERTAINMENT mobile apps" OR DE "IPHONE mobile apps" OR DE "MOBILE apps in business" OR DE "MOBILE apps in education" OR DE "NAVIGATION & travel mobile apps" OR DE "ONLINE dating mobile apps" OR DE "PHYSICAL fitness mobile apps" OR DE "READING mobile apps" OR DE "SHOPPING mobile apps" OR DE "SOCIAL networking mobile apps" OR DE "TABLET mobile apps" OR DE "WEATHER forecasting mobile apps" | 19,449 |
| S48 | DE "INTERNET" OR DE "ART & the Internet" OR DE "CONTENT filters (Computer science)" OR DE "CYBERSQUATTING" OR DE "DANCE & the Internet" OR DE "DIGITAL footprint" OR DE "HISPANIC Americans & Internet" OR DE "INTERNET & activism" OR DE "INTERNET & children" OR DE "INTERNET & older people" OR DE "INTERNET & teenagers" OR DE "INTERNET & terrorism" OR DE "INTERNET & women" OR DE "INTERNET & youth" OR DE "INTERNET access" OR DE "INTERNET celebrities" OR DE "INTERNET content" OR DE "INTERNET entertainment" OR DE "INTERNET friendship" OR DE "INTERNET in education" OR DE "INTERNET in higher education" OR DE "INTERNET in real estate business" OR DE "INTERNET in school libraries" OR DE "INTERNET protocols" OR DE "INTERNET traffic" OR DE "INTERNET usage monitoring" OR DE "LANGUAGE & the Internet" OR DE "LIBRARIES & the Internet" OR DE "LINK spam (Internet)" OR DE "LITERATURE & the Internet" OR DE "MUSEUMS & the Internet" OR DE "MUSIC & the Internet" OR DE "ONLINE ministry" OR DE "POETRY & the Internet" OR DE "SEX on the Internet" OR DE "SEXISM on the Internet" OR DE "THEATER & the Internet" OR DE "VIRTUAL communities" OR DE "WAIS (Information retrieval system)" OR DE "WIRELESS Internet" | 112,651 |
| S47 | DE "ARTIFICIAL intelligence in medicine" | 470 |
| S46 | DE "MIXED reality" OR DE "AUGMENTED reality" | 4,594 |
| S45 | DE "HEALTH information technology" OR DE "COMPUTERS in medicine" OR DE "INTERNET in medicine" | 10,110 |
| S44 | TI "mixed realit*" OR AB "mixed realit*" | 730 |
| S43 | TI virtual OR AB virtual | 136,856 |
| S42 | TI "augmented realit*" OR AB "augmented realit*" | 4,926 |
| S41 | TI internet* OR AB internet* | 275,442 |
| S40 | TI online OR AB online | 405,709 |
| S39 | TI app OR AB app | 46,692 |
| S38 | TI "mobile healthcare" OR AB "mobile healthcare" | 173 |
| S37 | TI "mobile application*" OR AB "mobile application*" | 7,367 |
| S36 | TI "remote rehabilitation" OR AB "remote rehabilitation" | 38 |
| S35 | TI teletherapy OR AB teletherapy | 358 |
| S34 | ( TI telerehabilitation OR AB telerehabilitation ) OR ( TI tele-rehabilitation OR AB tele-rehabilitation ) | 694 |
| S33 | TI "remote consultation*" OR AB "remote consultation*" | 257 |
| S32 | ( TI telemonitor* OR AB telemonitor* ) OR ( TI tele-monitor* OR AB tele-monitor* ) | 1,194 |
| S31 | ( TI teleconsultation OR AB teleconsultation ) OR ( TI tele-consultation OR AB tele-consultation ) | 969 |
| S30 | TI telecommunication* OR AB telecommunication* | 55,058 |
| S29 | ( TI telecare OR AB telecare ) OR ( TI tele-care OR AB tele-care ) | 765 |
| S28 | ( TI telehealth OR AB telehealth ) OR ( TI tele-health OR AB tele-health ) | 5,576 |
| S27 | ( TI ehealth OR AB ehealth ) OR ( TI e-health OR AB e-health ) | 3,721 |
| S26 | TI "mobile phone" OR AB "mobile phone" | 12,928 |
| S25 | ( TI mhealth OR AB mhealth ) OR ( TI m-health OR AB m-health ) | 2,280 |
| S24 | ( TI telemedicine OR AB telemedicine ) OR ( TI tele-medicine OR AB tele-medicine ) | 9,455 |
| S23 | TI wearable* OR AB wearable* | 18,482 |
| S22 | TI "monitoring sensor*" OR AB "monitoring sensor*" | 594 |
| S21 | TI "monitoring device*" OR AB "monitoring device*" | 3,188 |
| S20 | ( TI "smart glasses" OR AB "smart glasses" ) OR ( TI smartglasses OR AB smartglasses ) | 254 |
| S19 | TI "self-help device*" OR AB "self-help device*" | 8 |
| S18 | TI "wireless technolog*" OR AB "wireless technolog*" | 4,110 |
| S17 | TI "neural network*" OR AB "neural network*" | 129,701 |
| S16 | TI "machine learning" OR AB "machine learning" | 63,968 |
| S15 | TI "artificial intelligence" OR AB "artificial intelligence" | 29,816 |
| S14 | TI "electronic communication" OR AB "electronic communication" | 2,098 |
| S13 | TI "information and communications" OR AB "information and communications" | 2,872 |
| S12 | TI informatics OR AB informatics | 9,320 |
| S11 | TI "information technolog*" OR AB "information technolog*" | 69,361 |
| S10 | TI digital OR AB digital | 338,522 |
| S9 | TI "smart device*" OR AB "smart device*" | 1,924 |
| S8 | TI smartphone* OR AB smartphone* | 31,139 |
| S7 | TI tablet OR AB tablet | 44,872 |
| S6 | TI "mobile device*" OR AB "mobile device*" | 14,580 |
| S5 | TI "spinal cord trauma" OR AB "spinal cord trauma" | 283 |
| S4 | TI tetraplegi* OR AB tetraplegi* | 1,870 |
| S3 | TI quadriplegi* OR AB quadriplegi* | 1,932 |
| S2 | TI paraplegi* OR AB paraplegi* | 5,979 |
| S1 | TI "spinal cord injur*" OR AB "spinal cord injur*" | 21,091 |

**Table 3.** Business Source Premier search strategy

| **#** | **Query** | **Results** |
| --- | --- | --- |
| S54 | S53 (Limiters - Published Date: 20100101-20220331; Language: English) | 40 |
| S53 | S51 AND S52 | 60 |
| S52 | S6 OR S7 OR S8 OR S9 OR S10 OR S11 OR S12 OR S13 OR S14 OR S15 OR S16 OR S17 OR S18 OR S19 OR S20 OR S21 OR S22 OR S23 OR S24 OR S25 OR S26 OR S27 OR S28 OR S29 OR S30 OR S31 OR S32 OR S33 OR S34 OR S35 OR S36 OR S37 OR S38 OR S39 OR S40 OR S41 OR S42 OR S43 OR S44 OR S45 OR S46 OR S47 OR S48 OR S49 OR S50 | 1,527,053 |
| S51 | S1 OR S2 OR S3 OR S4 OR S5 | 970 |
| S50 | DE "ARTIFICIAL intelligence" | 35,021 |
| S49 | DE "INTERNET" | 82,885 |
| S48 | DE "WEB services" | 5,813 |
| S47 | DE "MOBILE app development" | 1,265 |
| S46 | DE "INFORMATION & communication technologies" | 9,486 |
| S45 | DE "CELL phones" OR DE "SMARTPHONES" | 50,353 |
| S44 | TI "mixed realit*" OR AB "mixed realit*" | 373 |
| S43 | TI virtual OR AB virtual | 80,066 |
| S42 | TI "augmented realit*" OR AB "augmented realit*" | 4,512 |
| S41 | TI internet* OR AB internet* | 362,217 |
| S40 | TI online OR AB online | 389,431 |
| S39 | TI app OR AB app | 59,103 |
| S38 | TI "mobile healthcare" OR AB "mobile healthcare" | 130 |
| S37 | TI "mobile application*" OR AB "mobile application*" | 10,627 |
| S36 | TI "remote rehabilitation" OR AB "remote rehabilitation" | 4 |
| S35 | TI teletherapy OR AB teletherapy | 28 |
| S34 | ( TI telerehabilitation OR AB telerehabilitation ) OR ( TI tele-rehabilitation OR AB tele-rehabilitation ) | 43 |
| S33 | TI "remote consultation*" OR AB "remote consultation*" | 30 |
| S32 | ( TI telemonitor* OR AB telemonitor* ) OR ( TI tele-monitor* OR AB tele-monitor* ) | 87 |
| S31 | ( TI teleconsultation OR AB teleconsultation ) OR ( TI tele-consultation OR AB tele-consultation ) | 49 |
| S30 | TI telecommunication* OR AB telecommunication* | 142,902 |
| S29 | ( TI telecare OR AB telecare ) OR ( TI tele-care OR AB tele-care ) | 86 |
| S28 | ( TI telehealth OR AB telehealth ) OR ( TI tele-health OR AB tele-health ) | 2,109 |
| S27 | ( TI ehealth OR AB ehealth ) OR ( TI e-health OR AB e-health ) | 1,383 |
| S26 | TI "mobile phone" OR AB "mobile phone" | 18,570 |
| S25 | ( TI mhealth OR AB mhealth ) OR ( TI m-health OR AB m-health ) | 424 |
| S24 | ( TI telemedicine OR AB telemedicine ) OR ( TI tele-medicine OR AB tele-medicine ) | 2,604 |
| S23 | TI wearable* OR AB wearable* | 8,498 |
| S22 | TI "monitoring sensor*" OR AB "monitoring sensor*" | 191 |
| S21 | TI "monitoring device*" OR AB "monitoring device*" | 1,302 |
| S20 | ( TI "smart glasses" OR AB "smart glasses" ) OR ( TI smartglasses OR AB smartglasses ) | 335 |
| S19 | TI "self-help device*" OR AB "self-help device*" | 1,091 |
| S18 | TI "wireless technolog*" OR AB "wireless technolog*" | 6,972 |
| S17 | TI "neural network*" OR AB "neural network*" | 26,837 |
| S16 | TI "machine learning" OR AB "machine learning" | 18,069 |
| S15 | TI "artificial intelligence" OR AB "artificial intelligence" | 24,968 |
| S14 | TI "electronic communication" OR AB "electronic communication" | 1,581 |
| S13 | TI "information and communications" OR AB "information and communications" | 3,367 |
| S12 | TI informatics OR AB informatics | 3,087 |
| S11 | TI "information technolog*" OR AB "information technolog*" | 142,954 |
| S10 | TI digital OR AB digital | 332,411 |
| S9 | TI "smart device*" OR AB "smart device*" | 1,366 |
| S8 | TI smartphone* OR AB smartphone* | 39,762 |
| S7 | TI tablet OR AB tablet | 24,558 |
| S6 | TI "mobile device*" OR AB "mobile device*" | 19,205 |
| S5 | TI "spinal cord trauma" OR AB "spinal cord trauma" | 2 |
| S4 | TI tetraplegi* OR AB tetraplegi* | 52 |
| S3 | TI quadriplegi* OR AB quadriplegi* | 193 |
| S2 | TI paraplegi* OR AB paraplegi* | 216 |
| S1 | TI "spinal cord injur*" OR AB "spinal cord injur*" | 560 |

**Table 4.** CINAHL search strategy

| **#** | **Query** | **Results** |
| --- | --- | --- |
| S59 | S58 (Limiters - Published Date: 20100101--20220331; English: Language) | 1,187 |
| S58 | S56 AND S57 | 1,808 |
| S57 | S7 OR S8 OR S9 OR S10 OR S11 OR S12 OR S13 OR S14 OR S15 OR S16 OR S17 OR S18 OR S19 OR S20 OR S21 OR S22 OR S23 OR S24 OR S25 OR S26 OR S27 OR S28 OR S29 OR S30 OR S31 OR S32 OR S33 OR S34 OR S35 OR S36 OR S37 OR S38 OR S39 OR S40 OR S41 OR S42 OR S43 OR S44 OR S45 OR S46 OR S47 OR S48 OR S49 OR S50 OR S51 OR S52 OR S53 OR S54 OR S55 | 472,096 |
| S56 | S1 OR S2 OR S3 OR S4 OR S5 OR S6 | 30,054 |
| S55 | (MH "Computer Simulation+") NOT (MH Molecular Docking Simulation) | 25,139 |
| S54 | (MH "Internet+") | 162,851 |
| S53 | MH mobile applications | 10,516 |
| S52 | (MH "telehealth+") | 31,418 |
| S51 | (MH "Wearable Sensors+") | 6,418 |
| S50 | (MH "Telecommunications+") NOT (MH Voice Mail) | 155,306 |
| S49 | (MH "artificial intelligence+") | 23,875 |
| S48 | MH medical informatics | 5,410 |
| S47 | (MH "digital technology+") | 4,895 |
| S46 | (MH "Computers, Hand-Held+") | 8,112 |
| S45 | TI "mixed realit*" OR AB "mixed realit*" | 149 |
| S44 | TI virtual OR AB virtual | 32,828 |
| S43 | TI "augmented realit*" OR AB "augmented realit*" | 897 |
| S42 | TI internet* OR AB internet* | 34,546 |
| S41 | TI online OR AB online | 87,649 |
| S40 | TI app OR AB app | 12,193 |
| S39 | TI "mobile healthcare" OR AB "mobile healthcare" | 110 |
| S38 | TI "mobile application*" OR AB "mobile application*" | 2,261 |
| S37 | TI "remote rehabilitation" OR AB "remote rehabilitation" | 24 |
| S36 | TI teletherapy OR AB teletherapy | 137 |
| S35 | ( TI telerehabilitation OR AB telerehabilitation ) OR ( TI tele-rehabilitation OR AB tele-rehabilitation ) | 716 |
| S34 | TI "remote consultation*" OR AB "remote consultation*" | 196 |
| S33 | ( TI telemonitor* OR AB telemonitor* ) OR ( TI tele-monitor* OR AB tele-monitor* ) | 1,014 |
| S32 | ( TI teleconsultation OR AB teleconsultation ) OR ( TI tele-consultation OR AB tele-consultation ) | 597 |
| S31 | TI telecommunication* OR AB telecommunication* | 1,185 |
| S30 | ( TI telecare OR AB telecare ) OR ( TI tele-care OR AB tele-care ) | 676 |
| S29 | ( TI telehealth OR AB telehealth ) OR ( TI tele-health OR AB tele-health ) | 6,483 |
| S28 | ( TI ehealth OR AB ehealth ) OR ( TI e-health OR AB e-health ) | 4,220 |
| S27 | TI "mobile phone" OR AB "mobile phone" | 2,942 |
| S26 | ( TI mhealth OR AB mhealth ) OR ( TI m-health OR AB m-health ) | 2,446 |
| S25 | ( TI telemedicine OR AB telemedicine ) OR ( TI tele-medicine OR AB tele-medicine ) | 7,229 |
| S24 | TI wearable* OR AB wearable* | 3,887 |
| S23 | TI "monitoring sensor*" OR AB "monitoring sensor*" | 55 |
| S22 | TI "monitoring device*" OR AB "monitoring device*" | 1,323 |
| S21 | ( TI "smart glasses" OR AB "smart glasses" ) OR ( TI smartglasses OR AB smartglasses ) | 55 |
| S20 | TI "self-help device*" OR AB "self-help device*" | 14 |
| S19 | TI "wireless technolog*" OR AB "wireless technolog*" | 304 |
| S18 | TI "neural network*" OR AB "neural network*" | 6,018 |
| S17 | TI "machine learning" OR AB "machine learning" | 9,363 |
| S16 | TI "artificial intelligence" OR AB "artificial intelligence" | 5,474 |
| S15 | TI "electronic communication" OR AB "electronic communication" | 472 |
| S14 | TI "information and communications" OR AB "information and communications" | 310 |
| S13 | TI informatics OR AB informatics | 11,237 |
| S12 | TI "information technolog*" OR AB "information technolog*" | 9,979 |
| S11 | TI digital OR AB digital | 44,184 |
| S10 | TI "smart device*" OR AB "smart device*" | 243 |
| S9 | TI smartphone* OR AB smartphone* | 7,857 |
| S8 | TI tablet OR AB tablet | 11,501 |
| S7 | TI "mobile device*" OR AB "mobile device*" | 2,154 |
| S6 | TI spinal cord trauma OR AB spinal cord trauma | 126 |
| S5 | (MH "spinal cord injuries+") | 22,995 |
| S4 | TI tetraplegi* OR AB tetraplegi* | 2,037 |
| S3 | TI quadriplegi* OR AB quadriplegi* | 1,440 |
| S2 | TI paraplegi* OR AB paraplegi* | 3,912 |
| S1 | TI "spinal cord injur*" OR AB "spinal cord injur*" | 18,385 |

**Table 5.** Library, Information Science & Technology Abstracts search strategy

| **#** | **Query** | **Results** |
| --- | --- | --- |
| S54 | S53 (Limiters - Publication Date: 20100101--20220331; English: Language) | 11 |
| S53 | S51 AND S52 | 20 |
| S52 | S6 OR S7 OR S8 OR S9 OR S10 OR S11 OR S12 OR S13 OR S14 OR S15 OR S16 OR S17 OR S18 OR S19 OR S20 OR S21 OR S22 OR S23 OR S24 OR S25 OR S26 OR S27 OR S28 OR S29 OR S30 OR S31 OR S32 OR S33 OR S34 OR S35 OR S36 OR S37 OR S38 OR S39 OR S40 OR S41 OR S42 OR S43 OR S44 OR S46 OR S47 OR S48 OR S49 OR S50 | 191,949 |
| S51 | S1 OR S2 OR S3 OR S4 OR S5 | 51 |
| S50 | DE "COMPUTERS" OR DE "INFORMATION & communication technologies" | 7,274 |
| S49 | DE "MEDICAL informatics" OR DE "INFORMATION storage & retrieval systems -- Medical care" OR DE "MEDICAL information storage & retrieval systems" | 4,777 |
| S48 | DE "ARTIFICIAL intelligence" OR DE "ARTIFICIAL neural networks" OR DE "ERROR-correcting codes" OR DE "EXPERT systems" OR DE "KNOWLEDGE representation (Information theory)" OR DE "MACHINE learning" OR DE "MACHINE translating" OR DE "NATURAL language processing" OR DE "QUESTION answering systems" OR DE "SEMANTIC networks (Information theory)" | 8,740 |
| S47 | DE "MOBILE computing" OR DE "MOBILE apps" | 3,196 |
| S46 | DE "INTERNET" OR DE "CONTENT filters (Computer science)" OR DE "CYBERSQUATTING" OR DE "INTERNET & youth" OR DE "INTERNET content" OR DE "INTERNET in education" OR DE "INTERNET in higher education" OR DE "INTERNET in school libraries" OR DE "INTERNET traffic" OR DE "LIBRARIES & the Internet" OR DE "LINK spam (Internet)" OR DE "LITERATURE & the Internet" OR DE "VIRTUAL communities" OR DE "WIRELESS Internet" | 27,005 |
| S45 | (MH "artificial intelligence+") | 2 |
| S44 | TI "mixed realit*" OR AB "mixed realit*" | 47 |
| S43 | TI virtual OR AB virtual | 10,853 |
| S42 | TI "augmented realit*" OR AB "augmented realit*" | 403 |
| S41 | TI internet* OR AB internet* | 43,186 |
| S40 | TI online OR AB online | 65,880 |
| S39 | TI app OR AB app | 4,504 |
| S38 | TI "mobile healthcare" OR AB "mobile healthcare" | 15 |
| S37 | TI "mobile application*" OR AB "mobile application*" | 675 |
| S36 | TI "remote rehabilitation" OR AB "remote rehabilitation" | 3 |
| S35 | TI teletherapy OR AB teletherapy | 1 |
| S34 | ( TI telerehabilitation OR AB telerehabilitation ) OR ( TI tele-rehabilitation OR AB tele-rehabilitation ) | 30 |
| S33 | TI "remote consultation*" OR AB "remote consultation*" | 18 |
| S32 | ( TI telemonitor* OR AB telemonitor* ) OR ( TI tele-monitor* OR AB tele-monitor* ) | 91 |
| S31 | ( TI teleconsultation OR AB teleconsultation ) OR ( TI tele-consultation OR AB tele-consultation ) | 34 |
| S30 | TI telecommunication* OR AB telecommunication* | 3,902 |
| S29 | ( TI telecare OR AB telecare ) OR ( TI tele-care OR AB tele-care ) | 23 |
| S28 | ( TI telehealth OR AB telehealth ) OR ( TI tele-health OR AB tele-health ) | 257 |
| S27 | ( TI ehealth OR AB ehealth ) OR ( TI e-health OR AB e-health ) | 907 |
| S26 | TI "mobile phone" OR AB "mobile phone" | 928 |
| S25 | ( TI mhealth OR AB mhealth ) OR ( TI m-health OR AB m-health ) | 589 |
| S24 | ( TI telemedicine OR AB telemedicine ) OR ( TI tele-medicine OR AB tele-medicine ) | 432 |
| S23 | TI wearable* OR AB wearable* | 414 |
| S22 | TI "monitoring sensor*" OR AB "monitoring sensor*" | 3 |
| S21 | TI "monitoring device*" OR AB "monitoring device*" | 67 |
| S20 | ( TI "smart glasses" OR AB "smart glasses" ) OR ( TI smartglasses OR AB smartglasses ) | 10 |
| S19 | TI "self-help device*" OR AB "self-help device*" | 21 |
| S18 | TI "wireless technolog*" OR AB "wireless technolog*" | 154 |
| S17 | TI "neural network*" OR AB "neural network*" | 1,008 |
| S16 | TI "machine learning" OR AB "machine learning" | 2,114 |
| S15 | TI "artificial intelligence" OR AB "artificial intelligence" | 3,540 |
| S14 | TI "electronic communication" OR AB "electronic communication" | 313 |
| S13 | TI "information and communications" OR AB "information and communications" | 649 |
| S12 | TI informatics OR AB informatics | 2,703 |
| S11 | TI "information technolog*" OR AB "information technolog*" | 13,439 |
| S10 | TI digital OR AB digital | 51,084 |
| S9 | TI "smart device*" OR AB "smart device*" | 77 |
| S8 | TI smartphone* OR AB smartphone* | 2,142 |
| S7 | TI tablet OR AB tablet | 1,466 |
| S6 | TI "mobile device*" OR AB "mobile device*" | 1,716 |
| S5 | TI "spinal cord trauma" OR AB "spinal cord trauma" | 75 |
| S4 | TI tetraplegi* OR AB tetraplegi* | 1 |
| S3 | TI quadriplegi* OR AB quadriplegi* | 9 |
| S2 | TI paraplegi* OR AB paraplegi* | 9 |
| S1 | TI "spinal cord injur*" OR AB "spinal cord injur*" | 34 |

**Table 6.** MEDLINE search strategy

| **#** | **Query** | **Results** |
| --- | --- | --- |
| S62 | S61 (Limiters - Date of Publication: 20100101--20220331; Language: English) | 2,255 |
| S61 | S59 AND S60 | 3,245 |
| S60 | S7 OR S8 OR S9 OR S10 OR S11 OR S12 OR S13 OR S14 OR S15 OR S16 OR S17 OR S18 OR S19 OR S20 OR S21 OR S22 OR S23 OR S24 OR S25 OR S26 OR S27 OR S28 OR S29 OR S30 OR S31 OR S32 OR S33 OR S34 OR S35 OR S36 OR S37 OR S38 OR S39 OR S40 OR S41 OR S42 OR S43 OR S44 OR S45 OR S46 OR S47 OR S48 OR S49 OR S50 OR S51 OR S52 OR S53 OR S54 OR S55 OR S56 OR S57 OR S58 | 1,254,625 |
| S59 | S1 OR S2 OR S3 OR S4 OR S5 OR S6 | 82,923 |
| S58 | MH virtual reality | 4,172 |
| S57 | MH augmented reality | 732 |
| S56 | (MH "Internet+") | 91,101 |
| S55 | MH mobile applications | 9,728 |
| S54 | MH telemedicine | 32,748 |
| S53 | MH wearable electronic devices | 5,773 |
| S52 | (MH "Telecommunications+") NOT (MH Radar OR MH Television OR MH Answering Services OR MH Telefacsimile OR (MH "Satellite Communications+")) | 96,831 |
| S51 | (MH "artificial intelligence+") | 140,428 |
| S50 | (MH "medical informatics+") | 484,377 |
| S49 | MH information technology | 647 |
| S48 | MH digital technology | 432 |
| S47 | MH telerehabilitation | 742 |
| S46 | MH computers, handheld | 3,952 |
| S45 | TI "mixed realit*" OR AB "mixed realit*" | 540 |
| S44 | TI virtual OR AB virtual | 76,264 |
| S43 | TI "augmented realit*" OR AB "augmented realit*" | 2,984 |
| S42 | TI internet* OR AB internet* | 61,308 |
| S41 | TI online OR AB online | 166,009 |
| S40 | TI app OR AB app | 34,456 |
| S39 | TI "mobile healthcare" OR AB "mobile healthcare" | 206 |
| S38 | TI "mobile application*" OR AB "mobile application*" | 3,585 |
| S37 | TI "remote rehabilitation" OR AB "remote rehabilitation" | 58 |
| S36 | TI teletherapy OR AB teletherapy | 1,405 |
| S35 | ( TI telerehabilitation OR AB telerehabilitation ) OR ( TI tele-rehabilitation OR AB tele-rehabilitation ) | 1,301 |
| S34 | TI "remote consultation*" OR AB "remote consultation*" | 477 |
| S33 | ( TI telemonitor* OR AB telemonitor* ) OR ( TI tele-monitor* OR AB tele-monitor* ) | 2,002 |
| S32 | ( TI teleconsultation OR AB teleconsultation ) OR ( TI tele-consultation OR AB tele-consultation ) | 1,681 |
| S31 | TI telecommunication* OR AB telecommunication* | 4,784 |
| S30 | ( TI telecare OR AB telecare ) OR ( TI tele-care OR AB tele-care ) | 738 |
| S29 | ( TI telehealth OR AB telehealth ) OR ( TI tele-health OR AB tele-health ) | 7,869 |
| S28 | ( TI ehealth OR AB ehealth ) OR ( TI e-health OR AB e-health ) | 5,951 |
| S27 | TI "mobile phone" OR AB "mobile phone" | 7,456 |
| S26 | ( TI mhealth OR AB mhealth ) OR ( TI m-health OR AB m-health ) | 4,869 |
| S25 | ( TI telemedicine OR AB telemedicine ) OR ( TI tele-medicine OR AB tele-medicine ) | 15,420 |
| S24 | TI wearable* OR AB wearable* | 18,386 |
| S23 | TI "monitoring sensor*" OR AB "monitoring sensor*" | 348 |
| S22 | TI "monitoring device*" OR AB "monitoring device*" | 4,313 |
| S21 | ( TI "smart glasses" OR AB "smart glasses" ) OR ( TI smartglasses OR AB smartglasses ) | 152 |
| S20 | TI "self-help device*" OR AB "self-help device*" | 67 |
| S19 | TI "wireless technolog*" OR AB "wireless technolog*" | 628 |
| S18 | TI "neural network*" OR AB "neural network*" | 70,825 |
| S17 | TI "machine learning" OR AB "machine learning" | 52,301 |
| S16 | TI "artificial intelligence" OR AB "artificial intelligence" | 17,600 |
| S15 | TI "electronic communication" OR AB "electronic communication" | 1,308 |
| S14 | TI "information and communications" OR AB "information and communications" | 498 |
| S13 | TI informatics OR AB informatics | 14,472 |
| S12 | TI "information technolog*" OR AB "information technolog*" | 14,018 |
| S11 | TI digital OR AB digital | 149,873 |
| S10 | TI "smart device*" OR AB "smart device*" | 943 |
| S9 | TI smartphone* OR AB smartphone* | 16,978 |
| S8 | TI tablet OR AB tablet | 56,386 |
| S7 | TI "mobile device*" OR AB "mobile device*" | 4,033 |
| S6 | TI "spinal cord trauma" OR AB "spinal cord trauma" | 903 |
| S5 | (MH "spinal cord injuries+") | 52,936 |
| S4 | TI tetraplegi* OR AB tetraplegi* | 4,486 |
| S3 | TI quadriplegi* OR AB quadriplegi* | 4,314 |
| S2 | TI paraplegi* OR AB paraplegi* | 17,551 |
| S1 | TI "spinal cord injur*" OR AB "spinal cord injur*" | 42,588 |

**Table 7.** PsycInfo search strategy

| **#** | **Query** | **Results** |
| --- | --- | --- |
| 60 | (tiab("spinal cord injur*") OR tiab(paraplegi*) OR tiab(quadriplegi*) OR tiab(tetraplegi*) OR tiab("spinal cord trauma")) AND (tiab("mobile device*") OR tiab(tablet) OR tiab(smartphone*) OR tiab("smart device*") OR tiab(digital) OR tiab("information technolog*") OR tiab(informatics) OR tiab("information and communications") OR tiab("electronic communication") OR tiab("artificial intelligence") OR tiab("machine learning") OR tiab("neural network*") OR tiab("wireless technolog*") OR tiab("self-help devices") OR tiab("smart glasses") OR tiab(smartglasses) OR tiab("monitoring device*") OR tiab("monitoring sensor*") OR tiab(wearable*) OR tiab(telemedicine) OR tiab(tele-medicine) OR tiab(mhealth) OR tiab(m-health) OR tiab("mobile phone") OR tiab(ehealth) OR tiab(e-health) OR tiab(telehealth) OR tiab(tele-health) OR tiab(telecare) OR tiab(tele-care) OR tiab(telecommunication*) OR tiab(teleconsultation) OR tiab(tele-consultation) OR tiab(telemonitor*) OR tiab(tele-monitor*) OR tiab("remote consultation*") OR tiab(telerehabilitation) OR tiab(tele-rehabilitation) OR tiab(teletherapy) OR tiab("remote rehabilitation") OR tiab("mobile application*") OR tiab("mobile healthcare") OR tiab(app) OR tiab(online) OR tiab(internet*) OR tiab("augmented realit*") OR tiab(virtual) OR tiab("mixed realit*")) AND (rtype.exact("Journal" OR "Peer Reviewed Journal" OR "Journal Article" OR "Dissertation" OR "Dissertation Abstract") AND stype.exact("Scholarly Journals" OR "Dissertations & Theses") AND la.exact("ENG") AND po.exact("Human") AND pd(20100101-20220331)) | 163 |
| 59 | (tiab("spinal cord injur*") OR tiab(paraplegi*) OR tiab(quadriplegi*) OR tiab(tetraplegi*) OR tiab("spinal cord trauma")) AND (tiab("mobile device*") OR tiab(tablet) OR tiab(smartphone*) OR tiab("smart device*") OR tiab(digital) OR tiab("information technolog*") OR tiab(informatics) OR tiab("information and communications") OR tiab("electronic communication") OR tiab("artificial intelligence") OR tiab("machine learning") OR tiab("neural network*") OR tiab("wireless technolog*") OR tiab("self-help devices") OR tiab("smart glasses") OR tiab(smartglasses) OR tiab("monitoring device*") OR tiab("monitoring sensor*") OR tiab(wearable*) OR tiab(telemedicine) OR tiab(tele-medicine) OR tiab(mhealth) OR tiab(m-health) OR tiab("mobile phone") OR tiab(ehealth) OR tiab(e-health) OR tiab(telehealth) OR tiab(tele-health) OR tiab(telecare) OR tiab(tele-care) OR tiab(telecommunication*) OR tiab(teleconsultation) OR tiab(tele-consultation) OR tiab(telemonitor*) OR tiab(tele-monitor*) OR tiab("remote consultation*") OR tiab(telerehabilitation) OR tiab(tele-rehabilitation) OR tiab(teletherapy) OR tiab("remote rehabilitation") OR tiab("mobile application*") OR tiab("mobile healthcare") OR tiab(app) OR tiab(online) OR tiab(internet*) OR tiab("augmented realit*") OR tiab(virtual) OR tiab("mixed realit*")) AND (rtype.exact("Journal" OR "Peer Reviewed Journal" OR "Journal Article" OR "Dissertation" OR "Dissertation Abstract") AND stype.exact("Scholarly Journals" OR "Dissertations & Theses") AND pd(20100101-20220331)) | 199 |
| 58 | (tiab("spinal cord injur*") OR tiab(paraplegi*) OR tiab(quadriplegi*) OR tiab(tetraplegi*) OR tiab("spinal cord trauma")) AND (tiab("mobile device*") OR tiab(tablet) OR tiab(smartphone*) OR tiab("smart device*") OR tiab(digital) OR tiab("information technolog*") OR tiab(informatics) OR tiab("information and communications") OR tiab("electronic communication") OR tiab("artificial intelligence") OR tiab("machine learning") OR tiab("neural network*") OR tiab("wireless technolog*") OR tiab("self-help devices") OR tiab("smart glasses") OR tiab(smartglasses) OR tiab("monitoring device*") OR tiab("monitoring sensor*") OR tiab(wearable*) OR tiab(telemedicine) OR tiab(tele-medicine) OR tiab(mhealth) OR tiab(m-health) OR tiab("mobile phone") OR tiab(ehealth) OR tiab(e-health) OR tiab(telehealth) OR tiab(tele-health) OR tiab(telecare) OR tiab(tele-care) OR tiab(telecommunication*) OR tiab(teleconsultation) OR tiab(tele-consultation) OR tiab(telemonitor*) OR tiab(tele-monitor*) OR tiab("remote consultation*") OR tiab(telerehabilitation) OR tiab(tele-rehabilitation) OR tiab(teletherapy) OR tiab("remote rehabilitation") OR tiab("mobile application*") OR tiab("mobile healthcare") OR tiab(app) OR tiab(online) OR tiab(internet*) OR tiab("augmented realit*") OR tiab(virtual) OR tiab("mixed realit*")) AND (stype.exact("Scholarly Journals" OR "Dissertations & Theses") AND pd(20100101-20220331)) | 199 |
| 57 | (tiab("spinal cord injur*") OR tiab(paraplegi*) OR tiab(quadriplegi*) OR tiab(tetraplegi*) OR tiab("spinal cord trauma")) AND (tiab("mobile device*") OR tiab(tablet) OR tiab(smartphone*) OR tiab("smart device*") OR tiab(digital) OR tiab("information technolog*") OR tiab(informatics) OR tiab("information and communications") OR tiab("electronic communication") OR tiab("artificial intelligence") OR tiab("machine learning") OR tiab("neural network*") OR tiab("wireless technolog*") OR tiab("self-help devices") OR tiab("smart glasses") OR tiab(smartglasses) OR tiab("monitoring device*") OR tiab("monitoring sensor*") OR tiab(wearable*) OR tiab(telemedicine) OR tiab(tele-medicine) OR tiab(mhealth) OR tiab(m-health) OR tiab("mobile phone") OR tiab(ehealth) OR tiab(e-health) OR tiab(telehealth) OR tiab(tele-health) OR tiab(telecare) OR tiab(tele-care) OR tiab(telecommunication*) OR tiab(teleconsultation) OR tiab(tele-consultation) OR tiab(telemonitor*) OR tiab(tele-monitor*) OR tiab("remote consultation*") OR tiab(telerehabilitation) OR tiab(tele-rehabilitation) OR tiab(teletherapy) OR tiab("remote rehabilitation") OR tiab("mobile application*") OR tiab("mobile healthcare") OR tiab(app) OR tiab(online) OR tiab(internet*) OR tiab("augmented realit*") OR tiab(virtual) OR tiab("mixed realit*")) AND pd(20100101-20220331) | 200 |
| 56 | (tiab("spinal cord injur*") OR tiab(paraplegi*) OR tiab(quadriplegi*) OR tiab(tetraplegi*) OR tiab("spinal cord trauma")) AND (tiab("mobile device*") OR tiab(tablet) OR tiab(smartphone*) OR tiab("smart device*") OR tiab(digital) OR tiab("information technolog*") OR tiab(informatics) OR tiab("information and communications") OR tiab("electronic communication") OR tiab("artificial intelligence") OR tiab("machine learning") OR tiab("neural network*") OR tiab("wireless technolog*") OR tiab("self-help devices") OR tiab("smart glasses") OR tiab(smartglasses) OR tiab("monitoring device*") OR tiab("monitoring sensor*") OR tiab(wearable*) OR tiab(telemedicine) OR tiab(tele-medicine) OR tiab(mhealth) OR tiab(m-health) OR tiab("mobile phone") OR tiab(ehealth) OR tiab(e-health) OR tiab(telehealth) OR tiab(tele-health) OR tiab(telecare) OR tiab(tele-care) OR tiab(telecommunication*) OR tiab(teleconsultation) OR tiab(tele-consultation) OR tiab(telemonitor*) OR tiab(tele-monitor*) OR tiab("remote consultation*") OR tiab(telerehabilitation) OR tiab(tele-rehabilitation) OR tiab(teletherapy) OR tiab("remote rehabilitation") OR tiab("mobile application*") OR tiab("mobile healthcare") OR tiab(app) OR tiab(online) OR tiab(internet*) OR tiab("augmented realit*") OR tiab(virtual) OR tiab("mixed realit*")) | 259 |
| 55 | tiab("mobile device*") OR tiab(tablet) OR tiab(smartphone*) OR tiab("smart device*") OR tiab(digital) OR tiab("information technolog*") OR tiab(informatics) OR tiab("information and communications") OR tiab("electronic communication") OR tiab("artificial intelligence") OR tiab("machine learning") OR tiab("neural network*") OR tiab("wireless technolog*") OR tiab("self-help devices") OR tiab("smart glasses") OR tiab(smartglasses) OR tiab("monitoring device*") OR tiab("monitoring sensor*") OR tiab(wearable*) OR tiab(telemedicine) OR tiab(tele-medicine) OR tiab(mhealth) OR tiab(m-health) OR tiab("mobile phone") OR tiab(ehealth) OR tiab(e-health) OR tiab(telehealth) OR tiab(tele-health) OR tiab(telecare) OR tiab(tele-care) OR tiab(telecommunication*) OR tiab(teleconsultation) OR tiab(tele-consultation) OR tiab(telemonitor*) OR tiab(tele-monitor*) OR tiab("remote consultation*") OR tiab(telerehabilitation) OR tiab(tele-rehabilitation) OR tiab(teletherapy) OR tiab("remote rehabilitation") OR tiab("mobile application*") OR tiab("mobile healthcare") OR tiab(app) OR tiab(online) OR tiab(internet*) OR tiab("augmented realit*") OR tiab(virtual) OR tiab("mixed realit*") | 222002 |
| 54 | tiab("spinal cord injur*") OR tiab(paraplegi*) OR tiab(quadriplegi*) OR tiab(tetraplegi*) OR tiab("spinal cord trauma") | 8050 |
| 53 | tiab("mixed realit*") | 199 |
| 52 | tiab(virtual) | 25114 |
| 51 | tiab("augmented realit*") | 1007 |
| 50 | tiab(internet*) | 39428 |
| 49 | tiab(online) | 99359 |
| 48 | tiab(app) | 7860 |
| 47 | tiab("mobile healthcare") | 30 |
| 46 | tiab("mobile application*") | 1130 |
| 45 | tiab("remote rehabilitation") | 5 |
| 44 | tiab(teletherapy) | 119 |
| 43 | tiab(tele-rehabilitation) | 36 |
| 42 | tiab(telerehabilitation) | 194 |
| 41 | tiab("remote consultation*") | 42 |
| 40 | tiab(tele-monitor*) | 20 |
| 39 | tiab(telemonitor*) | 189 |
| 38 | tiab(tele-consultation) | 8 |
| 37 | tiab(teleconsultation) | 147 |
| 36 | tiab(telecommunication*) | 1932 |
| 35 | tiab(tele-care) | 9 |
| 34 | tiab(telecare) | 206 |
| 33 | tiab(tele-health) | 50 |
| 32 | tiab(telehealth) | 2069 |
| 31 | tiab(e-health) | 913 |
| 30 | tiab(ehealth) | 871 |
| 29 | tiab("mobile phone") | 2610 |
| 28 | tiab(m-health) | 111 |
| 27 | tiab(mhealth) | 912 |
| 26 | tiab(tele-medicine) | 12 |
| 25 | tiab(telemedicine) | 1808 |
| 24 | tiab(wearable*) | 1578 |
| 23 | tiab("monitoring sensor*") | 20 |
| 22 | tiab("monitoring device*") | 367 |
| 21 | tiab(smartglasses) | 4 |
| 20 | tiab("smart glasses") | 26 |
| 19 | tiab("self-help devices") | 2 |
| 18 | tiab("wireless technolog*") | 131 |
| 17 | tiab("neural network*") | 17488 |
| 16 | tiab("machine learning") | 7515 |
| 15 | tiab("artificial intelligence") | 3957 |
| 14 | tiab("electronic communication") | 515 |
| 13 | tiab("information and communications") | 559 |
| 12 | tiab(informatics) | 1204 |
| 11 | tiab("information technolog*") | 7731 |
| 10 | tiab(digital) | 26303 |
| 9 | tiab("smart device*") | 148 |
| 8 | tiab(smartphone*) | 5294 |
| 7 | tiab(tablet) | 5036 |
| 6 | tiab("mobile device*") | 2308 |
| 5 | tiab("spinal cord trauma") | 71 |
| 4 | tiab(tetraplegi*) | 377 |
| 3 | tiab(quadriplegi*) | 551 |
| 2 | tiab(paraplegi*) | 1286 |
| 1 | tiab("spinal cord injur*") | 6417 |

**Table 8.** Scopus search strategy

| **#** | **Query** | **Results** |
| --- | --- | --- |
| 60 | #58 OR #59 | 1,567 |
| 59 | #57 AND (PUBDATETXT ( "january 2022" OR "february 2022" OR "march 2022" ) ) | 37 |
| 58 | #57 AND ( LIMIT-TO ( PUBYEAR , 2021 ) OR LIMIT-TO ( PUBYEAR , 2020 ) OR LIMIT-TO ( PUBYEAR , 2019 ) OR LIMIT-TO ( PUBYEAR , 2018 ) OR LIMIT-TO ( PUBYEAR , 2017 ) OR LIMIT-TO ( PUBYEAR , 2016 ) OR LIMIT-TO ( PUBYEAR , 2015 ) OR LIMIT-TO ( PUBYEAR , 2014 ) OR LIMIT-TO ( PUBYEAR , 2013 ) OR LIMIT-TO ( PUBYEAR , 2012 ) OR LIMIT-TO ( PUBYEAR , 2011 ) OR LIMIT-TO ( PUBYEAR , 2010 ) ) AND ( LIMIT-TO ( LANGUAGE , "English" ) ) AND ( LIMIT-TO ( DOCTYPE , "ar" ) OR LIMIT-TO ( DOCTYPE , "cp" ) OR LIMIT-TO ( DOCTYPE , "re" ) ) | 1,530 |
| 57 | #56 AND ( LIMIT-TO ( DOCTYPE , "ar" ) OR LIMIT-TO ( DOCTYPE , "cp" ) OR LIMIT-TO ( DOCTYPE , "re" ) ) AND ( LIMIT-TO ( LANGUAGE , "English" ) ) | 2,081 |
| 56 | #54 AND #55 | 2,352 |
| 55 | #6 OR #7 OR #8 OR #9 OR #10 OR #11 OR #12 OR #13 OR #14 OR #15 OR #16 OR #17 OR #18 OR #19 OR #20 OR #21 OR #22 OR #23 OR #24 OR #25 OR #26 OR #27 OR #28 OR #29 OR #30 OR #31 OR #32 OR #33 OR #34 OR #35 OR #36 OR #37 OR #38 OR #39 OR #40 OR #41 OR #42 OR #43 OR #44 OR #45 OR #46 OR #47 OR #48 OR #49 OR #50 OR #51 OR #52 OR #53 | 3,737,433 |
| 54 | #1 OR #2 OR #3 OR #4 OR #5 | 76,375 |
| 53 | TITLE-ABS ( "mixed realit*" ) | 5,561 |
| 52 | TITLE-ABS ( virtual ) | 457,177 |
| 51 | TITLE-ABS ( "augmented realit*" ) | 30,527 |
| 50 | TITLE-ABS ( internet* ) | 458,056 |
| 49 | TITLE-ABS ( online ) | 645,244 |
| 48 | TITLE-ABS ( app ) | 68,067 |
| 47 | TITLE-ABS ( "mobile healthcare" ) | 997 |
| 46 | TITLE-ABS ( "mobile application*" ) | 32,798 |
| 45 | TITLE-ABS ( "remote rehabilitation" ) | 147 |
| 44 | TITLE-ABS ( teletherapy ) | 1,756 |
| 43 | TITLE-ABS ( tele-rehabilitation ) | 471 |
| 42 | TITLE-ABS ( telerehabilitation ) | 1,541 |
| 41 | TITLE-ABS ( "remote consultation*" ) | 661 |
| 40 | TITLE-ABS ( tele-monitor* ) | 474 |
| 39 | TITLE-ABS ( telemonitor* ) | 2,875 |
| 38 | TITLE-ABS ( tele-consultation ) | 246 |
| 37 | TITLE-ABS ( teleconsultation ) | 1,646 |
| 36 | TITLE-ABS ( telecommunication* ) | 94,622 |
| 35 | TITLE-ABS ( tele-care ) | 88 |
| 34 | TITLE-ABS ( telecare ) | 1,602 |
| 33 | TITLE-ABS ( tele-health ) | 423 |
| 32 | TITLE-ABS ( telehealth ) | 9,212 |
| 31 | TITLE-ABS ( e-health ) | 8,461 |
| 30 | TITLE-ABS ( ehealth ) | 6,250 |
| 29 | TITLE-ABS ( "mobile phone" ) | 52,868 |
| 28 | TITLE-ABS ( m-health ) | 1,315 |
| 27 | TITLE-ABS ( mhealth ) | 6,225 |
| 26 | TITLE-ABS ( tele-medicine ) | 452 |
| 25 | TITLE-ABS ( telemedicine ) | 23,040 |
| 24 | TITLE-ABS ( wearable* ) | 69,656 |
| 23 | TITLE-ABS ( "monitoring sensor*" ) | 2,917 |
| 22 | TITLE-ABS ( "monitoring device*" ) | 11,487 |
| 21 | TITLE-ABS ( smartglasses ) | 135 |
| 20 | TITLE-ABS ( "smart glasses" ) | 1,203 |
| 19 | TITLE-ABS ( "self-help devices" ) | 79 |
| 18 | TITLE-ABS ( "wireless technolog*" ) | 12,830 |
| 17 | TITLE-ABS ( "neural network*" ) | 583,772 |
| 16 | TITLE-ABS ( "machine learning" ) | 267,256 |
| 15 | TITLE-ABS ( "artificial intelligence" ) | 122,269 |
| 14 | TITLE-ABS ( "electronic communication" ) | 5,606 |
| 13 | TITLE-ABS ( "information and communications" ) | 64,828 |
| 12 | TITLE-ABS ( informatics ) | 38,222 |
| 11 | TITLE-ABS ( "information technolog*" ) | 110,957 |
| 10 | TITLE-ABS ( digital ) | 959,397 |
| 9 | TITLE-ABS ( "smart device*" ) | 11,725 |
| 8 | TITLE-ABS ( smartphone* ) | 71,058 |
| 7 | TITLE-ABS ( tablet ) | 114,576 |
| 6 | TITLE-ABS ( "mobile device*" ) | 68,365 |
| 5 | TITLE-ABS ( "spinal cord trauma" ) | 1,106 |
| 4 | TITLE-ABS ( tetraplegi* ) | 5,551 |
| 3 | TITLE-ABS ( quadriplegi* ) | 5,667 |
| 2 | TITLE-ABS ( paraplegi* ) | 22,304 |
| 1 | TITLE-ABS ( "spinal cord injur*" ) | 51,175 |

**Table 9.** Web of Science

| **#** | **Query** | **Results** |
| --- | --- | --- |
| 59 | #58 AND English (Languages) | 1,596 |
| 58 | #57 AND Articles OR Review Articles OR Early Access OR Book Chapters OR Proceedings Papers (Document Types) | 1,612 |
| 57 | #56 AND Timespan: 2010-01-01 to 2022-03-31 (Publication Date) | 1,646 |
| 56 | #55 AND #54 | 1,995 |
| 55 | #53 OR #52 OR #51 OR #50 OR #49 OR #48 OR #47 OR #46 OR #45 OR #44 OR #43 OR #42 OR #41 OR #40 OR #39 OR #38 OR #37 OR #36 OR #35 OR #34 OR #33 OR #32 OR #31 OR #30 OR #29 OR #28 OR #27 OR #26 OR #25 OR #24 OR #23 OR #22 OR #21 OR #20 OR #19 OR #18 OR #17 OR #16 OR #15 OR #14 OR #13 OR #12 OR #11 OR #10 OR #9 OR #8 OR #7 OR #6 | 1,701,201 |
| 54 | #1 OR #2 OR #3 OR #4 OR #5 | 59,057 |
| 53 | TI=("mixed realit*") OR AB=("mixed realit*") | 1,384 |
| 52 | TI=(virtual) OR AB=(virtual) | 194,067 |
| 51 | TI=("augmented realit*") OR AB=("augmented realit*") | 8,514 |
| 50 | TI=(internet*) OR AB=(internet*) | 178,632 |
| 49 | TI=(online) OR AB=(online) | 372,648 |
| 48 | TI=(app) OR AB=(app) | 46,216 |
| 47 | TI=("mobile healthcare") OR AB=("mobile healthcare") | 337 |
| 46 | TI=("mobile application*") OR AB=("mobile application*") | 9,664 |
| 45 | TI=("remote rehabilitation") OR AB=("remote rehabilitation") | 65 |
| 44 | TI=(teletherapy) OR AB=(teletherapy) | 925 |
| 43 | TI=(tele-rehabilitation) OR AB=(tele-rehabilitation) | 258 |
| 42 | TI=(telerehabilitation) OR AB=(telerehabilitation) | 1,187 |
| 41 | TI=("remote consultation*") OR AB=("remote consultation*") | 442 |
| 40 | TI=(tele-monitor*) OR AB=(tele-monitor*) | 216 |
| 39 | TI=(telemonitor*) OR AB=(telemonitor*) | 2,041 |
| 38 | TI=(tele-consultation) OR AB=(tele-consultation) | 117 |
| 37 | TI=(teleconsultation) OR AB=(teleconsultation) | 1,137 |
| 36 | TI=(telecommunication*) OR AB=(telecommunication*) | 31,006 |
| 35 | TI=(tele-care) OR AB=(tele-care) | 36 |
| 34 | TI=(telecare) OR AB=(telecare) | 853 |
| 33 | TI=(tele-health) OR AB=(tele-health) | 248 |
| 32 | TI=(telehealth) OR AB=(telehealth) | 7,884 |
| 31 | TI=(e-health) OR AB=(e-health) | 3,551 |
| 30 | TI=(ehealth) OR AB=(ehealth) | 3,352 |
| 29 | TI=("mobile phone") OR AB=("mobile phone") | 13,717 |
| 28 | TI=(m-health) OR AB=(m-health) | 595 |
| 27 | TI=(mhealth) OR AB=(mhealth) | 4,388 |
| 26 | TI=(tele-medicine) OR AB=(tele-medicine) | 207 |
| 25 | TI=(telemedicine) OR AB=(telemedicine) | 15,501 |
| 24 | TI=(wearable*) OR AB=(wearable*) | 35,319 |
| 23 | TI=("monitoring sensor*") OR AB=("monitoring sensor*") | 1,129 |
| 22 | TI=("monitoring device*") OR AB=("monitoring device*") | 5,481 |
| 21 | TI=(smartglasses) OR AB=(smartglasses) | 46 |
| 20 | TI=("smart glasses") OR AB=("smart glasses") | 307 |
| 19 | TI=("self-help devices") OR AB=("self-help devices") | 48 |
| 18 | TI=("wireless technolog*") OR AB=("wireless technolog*") | 3,148 |
| 17 | TI=("neural network*") OR AB=("neural network*") | 249,400 |
| 16 | TI=("machine learning") OR AB=("machine learning") | 125,323 |
| 15 | TI=("artificial intelligence") OR AB=("artificial intelligence") | 49,321 |
| 14 | TI=("electronic communication") OR AB=("electronic communication") | 2,537 |
| 13 | TI=("information and communications") OR AB=("information and communications") | 2,859 |
| 12 | TI=(informatics) OR AB=(informatics) | 14,442 |
| 11 | TI=("information technolog*") OR AB=("information technolog*") | 37,597 |
| 10 | TI=(digital) OR AB=(digital) | 411,654 |
| 9 | TI=("smart device*") OR AB=("smart device*") | 4,168 |
| 8 | TI=(smartphone*) OR AB=(smartphone*) | 31,911 |
| 7 | TI=(tablet) OR AB=(tablet) | 65,786 |
| 6 | TI=("mobile device*") OR AB=("mobile device*") | 18,541 |
| 5 | TI=("spinal cord trauma") OR AB=("spinal cord trauma") | 773 |
| 4 | TI=(tetraplegi*) OR AB=(tetraplegi*) | 3,701 |
| 3 | TI=(quadriplegi*) OR AB=(quadriplegi*) | 3,429 |
| 2 | TI=(paraplegi*) OR AB=(paraplegi*) | 14,347 |
| 1 | TI=("spinal cord injur*") OR AB=("spinal cord injur*") | 42,530 |
