# Supplementary material for: Mobile Health Self-management Support for Spinal Cord Injury: Systematic Literature Review
Source: JMIR Mhealth Uhealth. 2023 Apr 26;11:e42679. doi: 10.2196/42679 (PMC10173031; doi:10.2196/42679)
Supplement: Multimedia Appendix 3 [file mhealth_v11i1e42679_app3.docx]

**Table S1.** Characteristics of the included publications (N=24).

| Study, publication year, and country | Aim | mHealth^a^ tool name | Primary technology | Self-management focus areas | Method | Sample size, N | Sex, n (%) | Key findings and conclusions |
| --- | --- | --- | --- | --- | --- | --- | --- | --- |
| Goodwin et al [53], 2021, United States | To use visualization techniques to understand the daily distributions of user interaction with a mobile pressure-mapping app for wheelchair users at risk of PI^b^ | AW-Shift^c^ | Pressure mat and mobile phone | PI management | Quasi-experimental study | 6 | Male: 5 (83); female: 1 (17) | The visualizations demonstrated that the system was successfully used for morning wheelchair setup and pressure spot checks throughout a 7-day period. Participant-indicated adherence to reminders to perform a weight shift was low, suggesting that other parts of the system (such as the live pressure mat) were more useful than the weight shift reminders. |
| Fizzotti et al [37], 2015, Italy | To describe a feasibility study of the introduction of a new modality to reinforce patient attitudes toward trunk rehabilitation and transform therapeutic exercises into captivating and educational games performed with a tablet | Ball Strike, Pop Flux | Tablet | Therapeutic exercise for hands, legs, or trunk | Quasi-experimental study | 15 | Male: 12 (80); female: 3 (20) | A significant improvement in trunk control was observed, and feedback was positive. |
| Olney et al [46], 2019, United States | To report the iterative redesign, feasibility, and usability of the CMAP^d^ system’s app used by veterans with SCI^e^ | CMAP | Mobile phone, pressure mat, and Raspberry Pi | PI management | Mixed methods study | 18 | Male: 17 (94); female: 1 (6) | A pressure-mapping system for determining appropriate seating equipment and positioning strategies for wheelchair users with SCI was presented. Usability interviews revealed that (1) any tool that can assist in prevention and monitoring of skin ulcers is deemed important; (2) the desired key features are present in the app; (3) the main barrier to app use was inconsistent functionality; and (4) when functioning as expected, the live pressure map was the central feature, with reminders to weight shift also of high importance. The survey found that power wheelchair users tended to score closer than manual wheelchair users to the positive response end ranges on 2 separate surveys. |
| Bizzarini et al [57], 2022, Italy | To propose an app and evaluate its feasibility with a pilot study in a clinical intervention | Fisiofriend | Mobile phone | PA^f^ promotion | Quasi-experimental study | 14 | Male: 14 (100) | The app was perceived to be pleasant and engaging and produce positive effects. Potentially useful app features for the intervention were reported. |
| Kryger et al [44], 2019, United States | To determine if the use of the iMHere^g^ system to support self-management for SCI would be associated with improved health outcomes over a 9-month period | iMHere | Mobile phone | Bladder management, PI management, and psychosocial support | Experimental study | 38 | Male: 25 (66); female: 13 (34) | A statistically significant reduction in urinary tract infections was observed. Psychosocial outcomes did not change significantly but met the threshold for clinical significance. Approximately 34 minutes per participant per month were needed on average to manage the system and provide technical support through this mHealth system. |
| Estes et al [36], 2015, Turkey | To evaluate the effectiveness of a wearable vibration glove for improving hand sensory functions in persons with partial SCI using passive haptic rehabilitation | MMT^h^ | Mobile device | Therapeutic exercise for hands, legs, or trunk | Experimental study | 7 | Male: 6 (86); female: 1 (14) | A statistically significant improvement in average Semmes-Weinstein scores for sensory functions in 8 points on the hand was observed in the experimental group after using the wearable vibration glove. |
| Wilroy et al [55], 2021, United States | To examine personal factors, secondary health conditions, and environmental factors as potential correlates of adherence to a home-based, thrice-weekly exercise program on a web-based application for wheelchair users | M2M^i^ | Tablet and wearable (watch) | PA promotion | Observational study (cross-sectional) | 28 | Male: 9 (32); female: 19 (68) | Ethnicity, community barriers, anxiety, depression, physical function, and self-efficacy were associated with a lower number of exercise video minutes. Pain intensity, pain interference, and age were associated with a higher number of exercise video minutes. |
| Khan and Phung [38], 2016, United States | To document the design and development of a preventive weight-shifting app to reduce the risk of PI in patients with SCI who are wheelchair users | NR^j^ | Mobile device | PI management | Technical note | N/A^k^ | N/A | The app provides information on the causes and prevention of PI. It also helps the user perform periodic skin inspections by providing step-by-step instructions with pictures to spot early signs of tissue breakdown. Therapists or caregivers can set up the frequency of weight shifting by setting notifications and reminders. |
| Wilde et al [40], 2016, United States | To evaluate the feasibility of a web-based intermittent catheter self-management intervention | NR | Mobile device | Bladder management | Mixed methods study | 29 | Male: 15 (52); female: 14 (48) | A statistically significant effect on self-management improvement on neurogenic bladder dysfunction was observed. The frequency of catheterizations, catheter-related self-efficacy and quality of life scores, urinary tract infection, and pain changed positively but not significantly. Intervention components, except for the forum, were rated highly by most participants for usefulness, satisfaction (desired information), and web-based usability. |
| Kongcharoen et al [43], 2019, Thailand | To develop a gamification smartphone app for leg physical therapy for SCI | NR | Mobile phone | Therapeutic exercise for hands, legs, or trunk | Quasi-experimental study | 10 | NR | A leg physical therapy app was developed with prominent gamification features. User engagement was tracked, and trophies were awarded with increased engagement. The app received a positive assessment by users. |
| Amann et al [48], 2020, Switzerland | To establish a co-design approach for developing a high-fidelity prototype app for the self-management of SCI, design the resulting prototype, and evaluate its usability | NR | Mobile phone | PI management | Mixed methods study | 4 | Male: 3 (75); female: 1 (25) | The co-design process resulted in a high-fidelity prototype. The final prototype included a combination of features to support people with SCI in the prevention of PI, namely, a smart camera, PI diary, expert consultation, reminders, and knowledge repository. A usability score of 78.5/100 was observed. |
| Amann et al [49], 2020, Switzerland | To identify the perceived benefits of a co-designed self-management app for SCI that could promote its uptake and to explore the factors that may impede adoption | NR | Mobile phone | PI management | Qualitative study | 27 | Male: 12 (44); female: 15 (55) | The app prototype was deemed potentially useful for supporting individuals with SCI in preventing PI as it acts as (1) a companion for newly injured individuals, (2) an emergency kit and motivational support, and (3) a guide for informal caregivers and family members. Potential adoption challenges include (1) challenges in motivating individuals to use the app, (2) concerns about the misuse and abuse of the app, and (3) organizational and maintenance challenges. |
| Wilde et al [58], 2015, United States | To provide urological practitioners with information about intermittent catheterization self-management using web-based materials and processes, including use of tables and figures | NR | Mobile device | Bladder management | Technical note | N/A | N/A | The intervention was introduced as a new and unique web-based urinary diary, which was modified for mobile phone use; 3 study nurse phone call contacts; and peer-led discussion forums. |
| Newman et al [45], 2019, United States | To develop educational content and pilot-test the use of a tablet, web-based content management platform, and videoconferencing for delivery of a peer-supported SCI self-management intervention using a community-engaged research approach | PHOENIX^l^ | Tablet | PI management, bladder management, and bowel management | Observational study (cross-sectional) | 10 | Male: 8 (80); female: 2 (20) | Educational content for the intervention was outlined. Participants were receptive and satisfied with the iPad, iTunes U platform, and video chat experience. Results showed that the iPad and iTunes U likely minimized usability issues with the technology. The use of FaceTime to facilitate video contact between the participants and peer navigators demonstrated satisfactory usability and acceptability. |
| Hiremath et al [42], 2019, United States | To use an mHealth-based PA measurement system to track the PA levels of individuals with SCI in the community and provide them with a behavior-sensitive, just-in-time adaptive intervention to improve their PA levels | PHIRE^m^ | Mobile phone and wearable (watch) | PA promotion | Quasi-experimental study | 20 | Male: 16 (80); female: 4 (20) | Demonstrated the use of an app for collecting PA level measurements to provide tailored feedback for individuals with SCI. Only 2 participants observed changes in energy expenditure during PA feedback with the intervention. During PA feedback with the intervention, a total of 11 participants showed higher levels of light-intensity PA, moderate-intensity PA, or both when compared with the baseline. |
| Shirai et al [52], 2020, Canada | To explore the experiences of individuals living with SCID^n^ on the use of PUT^o^, a mobile educational app for PI prevention and management | PUT | Tablet | PI management | Qualitative study | 9 | Male: 5 (56); female: 4 (44) | Experiences fell into four themes: (1) general strengths and weaknesses; (2) challenges resulting from a heterogeneous user base; (3) importance of prevention and use implication for enhanced quality of life; and (4) recommendations for improvement, including the need for additional information, improved visuals, and accessibility. These apps should be introduced early in rehabilitation to motivate users to prevent PI. Incorporating pictures is recommended. |
| Potiart et al [51], 2020, Thailand | To develop and evaluate the effectiveness of a smartphone app to assist the self-management of intermittent urinary catheter users | Punsook | Mobile phone | Bladder management and pain management | Quasi-experimental study | 35 | Male: 18 (51); female: 17 (49) | No statistically significant changes in bladder control were observed. Most participants reported that the app was pleasant. They liked the simplicity, ease of use, and accessibility and had interest in the program. |
| Mortenson et al [41], 2018, Canada | To outline a proposed study that will evaluate the efficacy of a self-management app in populations with SCI | SCI Health Storylines | Tablet | Bladder management, bowel management, PI management, spasticity management, autonomic dysreflexia management, PA promotion, pain management, psychosocial support, and medicating and dieting | Mixed methods study | N/A | N/A | A study to evaluate the efficacy of a self-management app in populations with SCI was outlined. If successful, this study will produce credible new knowledge describing multiple outcomes that people with SCI realize from an app-based self-management intervention. |
| Singh et al [47], 2019, Canada | To explore patient perspectives on the usability of the offered self-management app | SCI Health Storylines | Tablet | PI management, bowel management, bladder management, spasticity management, pain management, psychosocial support, and medicating and dieting | Mixed methods study | 20 | Male: 17 (85); female: 3 (15) | Three themes emerged from the qualitative analysis as being important to users: (1) the app being easy to adopt and compatible with assistive technologies, (2) the app being intuitive to navigate, and (3) the app offering flexibility and control over personal data. The app received above-average mean System Usability Scale scores. |
| Mortenson et al [59], 2019, Canada | To describe stakeholder perspectives on the development of a functional app to facilitate self-management skills needed to prevent secondary complications following recent SCI during inpatient rehabilitation | SCI Health Storylines | N/A | PI management, bowel management, bladder management, medicating and dieting, psychosocial support, and pain management | Qualitative study | 75 | Male: 20 (27); female: 55 (73) | Three themes emerged: (1) being individualized and user-friendly, (2) targeting goals to promote self-management, and (3) increasing participation and support seeking to facilitate lifestyle change. |
| MacGillivray et al [50], 2020, Canada | To determine the feasibility of implementing and evaluating a self-management app for SCI during inpatient rehabilitation and following community discharge | SCI Storylines | Tablet | PI management, bowel management, bladder management, PA promotion, pain management, and autonomic dysreflexia management | Quasi-experimental study | 20 | Male: 17 (85); female: 3 (15) | Feasibility indicators support a larger clinical trial during inpatient rehabilitation. However, challenges with retention and adherence following community discharge were observed. Bowel management confidence significantly improved over time, and there were trends in improvement for bladder, autonomic dysreflexia, and pain management confidence between admission and discharge. |
| Hoevenaars et al [54], 2021, the Netherlands | To develop an mHealth app that promotes a healthy lifestyle for wheelchair users with SCI or lower limb amputation and explore its usability, feasibility, and effectiveness | WHEELS^p^ | Tablet and mobile phone | PA promotion, psychosocial support, sleep management, and medicating and dieting | Mixed methods study | 14 | Male: 9 (64); female: 5 (36) | A total of 16 behavior change strategies were built into the app to change the PA, dietary, sleep, and relaxation behaviors of wheelchair users. User feedback indicated a varied experience using the app. Usability scores were positive, and the app was deemed feasible to deploy at a larger scale. |
| Cole et al [56], 2019, United States | To use a participatory action research approach to translate a theory-based intervention to be delivered via the web to individuals with SCI | WOWii^q^ | Mobile device | PA promotion | Qualitative study | 7 | Male: 4 (40); female: 3 (30) | The substantially redesigned website offers an easier-to-navigate platform for people with SCI with greater functionality that delivers information using a module format with less text and short video segments and presents more resources. |
| Wang et al [39], 2016, China and Belgium | To outline the development of a garment designed to support posture monitoring for rehabilitation training | Zishi | Wearable (garment), mobile phone, and tablet | Shoulder posture monitoring | Technical note | NS^r^ | NS | A garment designed to support posture monitoring for the purposes of rehabilitation training is presented. It consists of a garment integrated with smart textiles and wearable electronics. It presents real-time feedback as a vibration delivered through the garment and visual and audio instructions through a mobile device. |

^a^mHealth: mobile health.

^b^PI: pressure injury.

^c^AW-Shift: Assisted Weight Shift.

^d^CMAP: Comprehensive Mobile Assessment of Pressure.

^e^SCI: spinal cord injury.

^f^PA: physical activity.

^g^iMHere: Interactive Mobile Health and Rehabilitation.

^h^MMT: Mobile Music Touch.

^i^M2M: Movement-to-Music.

^j^NR: not reported.

^k^N/A: not applicable.

^l^PHOENIX: Peer-Supported Health Outreach, Education, and Information Exchange.

^m^PHIRE: Personal Health Informatics and Rehabilitation Engineering.

^n^SCID: SCI disorder.

^o^PUT: Pressure Ulcer Target.

^p^WHEELS: Wheelchair Exercise and Lifestyle Study.

^q^WOWii: Workout on Wheels internet intervention.

^r^NS: not specified.

**Table S2.** Characteristics of the included mobile health (mHealth) tools (n=19).

| mHealth tool name, citation, and country availability | Primary technology | Paired technologies | Operating system | Other device requirements | Design approach | Self-management focus areas | Description | Assessment types | Methods | Results |
| --- | --- | --- | --- | --- | --- | --- | --- | --- | --- | --- |
| AW-Shift^a^ [53], United States | Mobile app | Pressure mat and mobile phone | Android and iOS | Internet connectivity, Bluetooth, and display | NR^b^ | Pressure injury management | Users sit on a pressure mat fitted onto their wheelchair’s seat, and a Raspberry Pi minicomputer transmits data to a smartphone for real-time pressure mat visualization and provides feedback to users. | NR | NR | N/A^c^ |
| Ball Strike, Pop Flux [37], Italy | Mobile app | Tablet | iOS | Camera and display | NR | Therapeutic exercise for hands, legs, or trunk | Users receive a personalized rehabilitation program, including traditional neurorehabilitation exercises practiced through a sequence of games. Users sit in front of a tablet, and their movements are projected onto the screen within the gaming environment. These games involve hitting moving colored balls while avoiding other balls and blowing soap bubbles while avoiding other objects. | Usability | Survey | High satisfaction reported by 53% of the participants for both apps |
| CMAP^d^ [46], United States | Mobile app | Mobile phone, pressure mat, and Raspberry Pi | iOS | Display, notifications, Bluetooth, and internet connectivity | User-centered design | Pressure injury management | Users personalize movement notifications with a companion app, sit on a pressure-sensitive seat fitted to a wheelchair, and review data visualizations of sitting habits. | Usability and user experience | SUS^e^ and User Experience Questionnaire | SUS: x̄=72.1 and s=19.07 (good); User Experience Questionnaire: x̄≥0.8 for each dimension (positive) |
| Fisiofriend [57], Italy | Mobile app | Mobile phone | Android | Accelerometer, audio, and display | User-centered design | Physical activity promotion | Users receive instructions for personalized exercises from multimedia resources. Users attach a mobile phone to their wrists to monitor movement in real time and receive feedback based on accelerometer data. Users can also collect points and medals with increased engagement. The app can also be personalized by health care professionals. | User experience | Survey | x̄≥4.84 for each dimension (positive) |
| iMHere^f^ [44], United States | Mobile app | Mobile phone | Android | Reminders, internet connectivity, camera, messaging, and display | User-centered design | Bladder management, pressure injury management, and psychosocial support | Users participate in several modules as needed: (1) medication management, including medication administration reminders, the ability to upload photos of the medications, and customizable descriptions of the purpose for taking them; (2) urinary and bowel program reminders, with a system for reporting concerning symptoms; (3) skin care tracking with photo capabilities to monitor for pressure injury and skin breakdown; (4) mood tracking with validated surveys; and (5) messaging to communicate with a clinician. | NR | NR | N/A |
| MMT^g^ [36], Turkey | Wearable (glove) | Mobile device | NR | Audio and Bluetooth | User-centered design | Therapeutic exercise for hands, legs, or trunk | Users play pentatonic songs on a piano wearing a glove with vibration motors that guides their fingers to press the appropriate keys. Users also wear the glove for 2 hours daily for at least 5 days weekly. | NR | NR | N/A |
| M2M^h^ [55], United States | Mobile app | Tablet and wearable (watch) | NR | Audio, display, and internet connectivity | NR | Physical activity promotion | Users watch and follow self-managed exercise video sessions and read health promotion information via a web-based application on a tablet. | NR | NR | N/A |
| NR [38], United States | Mobile app | Mobile device | iOS | Notifications, reminders, accelerometer, cloud storage, internet connectivity, and display | NR | Pressure injury management | Users read information on the causes and prevention of pressure injury; receive instructions for periodic skin inspections, weight shifting, and tilting; and receive notifications set by therapists, caregivers, or the app to perform these activities based on tracked parameters. | NR | NR | N/A |
| NR [40,58], United States | Web-based (mobile-optimized) | Mobile device | All | Display and internet connectivity | User-centered design | Bladder management | Users access a web-based program with information related to SCI^i^ self-management and intermittent catheterization, a urinary diary, and a peer-led discussion forum and are supported by 3 consultations with a nurse as needed. | Usability | Survey | High for web-based information |
| NR [43], Thailand | Mobile app | Mobile phone | Android | Camera, accelerometer, internet connectivity, display, and reminders | NR | Therapeutic exercise for hands, legs, or trunk | Users attach a smartphone to walking support equipment to capture data and assess and receive weekly reports on their progress doing physical therapy exercises. Users can also personalize exercise reminders. | User experience | Survey | x̄=4.40 (good) |
| NR [48,49], Switzerland | Mobile app | Mobile phone | Android | Camera, messaging, reminders, display, internet connectivity, and audio | Participatory design | Pressure injury management | Users take photos of pressure injury, maintain a multimedia diary, consult with experts via text-or audio-based messaging or both, receive reminders about how to avoid injury, and learn more about pressure injury using a knowledge repository. | Usability | SUS, interview, and field study | SUS: x̄=78.5 (good); interview: high; field study: high |
| PHOENIX^j^ [45], United States | Mobile app | Tablet | iOS | Audio, display, internet connectivity, camera, and messaging | Participatory design | Pressure injury management, bladder management, and bowel management | Users access multimedia educational content on prevention of secondary conditions such as pressure injury and urinary tract infections via an app on a tablet. | Usability | Field study | iPad device: x̄=4.47 (favorable); iTunes U content: x̄=4.51 (favorable); video chat: x̄=4.53 (favorable) |
| PHIRE^k^ [42], United States | Mobile app | Mobile phone and wearable (watch) | Android | Accelerometer, display, notifications, and Bluetooth | NR | Physical activity promotion | Users wear a smartwatch and fit a rotation monitor to their wheelchair that streams ecological momentary assessment data to their smartphone. Users receive notifications to provide intermittent feedback about their activities. | NR | NR | N/A |
| PUT^l^ [52], Canada | Mobile app | Tablet | iOS | Display | User-centered design | Pressure injury management | Users access information about pressure injury via an app without the need for internet access. | User experience | Interviews | Need for additional information, improved visuals, and accessibility |
| Punsook [51], Thailand | Mobile app | Mobile phone | NR | Display and internet connectivity | User-centered design | Bladder management and pain management | Users maintain a voiding diary, learn more about bowel management using a knowledge guidebook and frequently asked questions repository, and can consult a physician for more information. | Usability and user experience | Survey | Usability: despite positive reviews after 1 month, most users found the app difficult to use after 2 months; user experience: very satisfied after 1 month and again after 3 months |
| SCI Health Storylines [41,47,50,59], Canada | Mobile app | Tablet | Android and iOS | Display | User-centered design | Bladder management, bowel management, pressure injury management, spasticity management, autonomic dysreflexia management, physical activity promotion, pain management, psychosocial support, and medicating and dieting | Users set self-management goals for bowel and bladder management, skin management, spasticity management, and daily exercise, as well as more acute topics, including urinary tract infections and autonomic dysreflexia, and have the option to track their progress using an app. | Usability and user testing | Usability: SUS; User testing: interviews, focus groups, and survey | Usability: x̄=78.1 (good) at discharge and x̄=71.6 (good) after 3 months; user testing: requires increased personalization features and support for achieving self-management goals and behavior change and increasing engagement |
| WHEELS^m^ [54], the Netherlands | Mobile app | Tablet and mobile phone | NR | Display, reminders, and internet connectivity | User-centered design | Physical activity promotion, psychosocial support, sleep management, and medicating and dieting | Wheelchair users personalize and follow an exercise program, nutrition plan, and sleep and relaxation exercises and participate in a community forum via an app. | NR | NR | N/A |
| WOWii^n^ [56], United States | Web-based (mobile-optimized) | Mobile device | All | Display and internet connectivity | Participatory design | Physical activity promotion | Users will have access to an exercise guide, exercise planning tasks, exercise tracking, peer resources, achievement tracking, a discussion forum, and a leaderboard in support of increasing their physical activity. | Usability | Interview | Improvements in design, content, and functionality and program delivery were suggested. |
| Zishi [39], China and Belgium | Mobile app | Wearable (garment), mobile phone, and tablet | Android | Display, notifications, audio, and Bluetooth | User-centered design | Shoulder posture monitoring | Users wear a garment with integrated smart textiles and wearable electronics and follow real-time visual guidance on posture using an app. | Usability | NS^o^ | Results forthcoming |

^a^AW-Shift: Assisted Weight Shift.

^b^NR: not reported.

^c^N/A: not applicable.

^d^CMAP: Comprehensive Mobile Assessment of Pressure.

^e^SUS: System Usability Scale.

^f^iMHere: Interactive Mobile Health and Rehabilitation.

^g^MMT: Mobile Music Touch.

^h^M2M: Movement-to-Music.

^i^SCI: spinal cord injury.

^j^PHOENIX: Peer-Supported Health Outreach, Education, and Information Exchange.

^k^PHIRE: Personal Health Informatics and Rehabilitation Engineering.

^l^PUT: Pressure Ulcer Target.

^m^WHEELS: Wheelchair Exercise and Lifestyle Study.

^n^WOWii: Workout on Wheels internet intervention.

^o^NS: not specified.
