# Supplementary material for: Mobile Health Self-management Support for Spinal Cord Injury: Systematic Literature Review
Source: JMIR Mhealth Uhealth. 2023 Apr 26;11:e42679. doi: 10.2196/42679 (PMC10173031; doi:10.2196/42679)
Supplement: Multimedia Appendix 5 [file mhealth_v11i1e42679_app5.docx]

## Multimedia Appendix 5

**Device requirements by self-management area, skills and support methods**

Table S1. Device requirements by self-management tasks, skills, support components and focus areas (N=19).

| Characteristics and citations | Internet connectivity | Bluetooth | Display | Camera | Notifications | Internet connectivity | Accelerometer | Audio | Reminders | Messaging | Cloud storage | Total coverage n (%) |
| --- | --- | --- | --- | --- | --- | --- | --- | --- | --- | --- | --- | --- |
| Self-management tasks |  |  |  |  |  |  |  |  |  |  |  |  |
| Medical management [1-19] | 3 [4, 7, 9] | 2 [5, 19] | 5 [2, 4, 7, 9, 19] | 3 [2, 4, 9] | 2 [7, 19] | 3 [4, 7, 9] | 2 [7, 9] | 2 [5, 19] | 3 [4, 7, 9] | 1 [4] | 1 [7] | 11 (100) |
| Role management [7, 8, 10-18, 20-24] | 9 [1, 6-8, 10-12, 17, 18, 21, 24] | 2 [1, 22] | 14 [1, 2, 6-8, 10-18, 20-24] | 3 [2, 10, 11, 21] | 2 [7, 22] | 9 [1, 6-8, 10-12, 17, 18, 21, 24] | 3 [7, 20, 22] | 4 [6, 10, 11, 20, 21] | 3 [7, 10, 11, 17] | 2 [10, 11, 21] | 1 [7] | 11 (100) |
| Emotional management [4, 13-15, 17] | 2 [4, 6] | —^a^ | 2 [4, 6] | 1 [4] | — | 2 [4, 6] | — | 1 [6] | 1 [4] | 1 [4] | — | 7 (64) |
| Self-management skills |  |  |  |  |  |  |  |  |  |  |  |  |
| Problem solving [3, 5, 6, 8, 10-12, 17, 18, 21, 23, 24] | 8 [3, 6, 8, 10-12, 17, 18, 21, 24] | 2 [3, 5] | 9 [3, 6, 8, 10-12, 17, 18, 21, 23, 24] | 2 [10, 11, 21] | 1 [3] | 8 [3, 6, 8, 10-12, 17, 18, 21, 24] | — | 4 [5, 6, 10, 11, 21] | 2 [10, 11, 17] | 2 [10, 11, 21] | — | 9 (82) |
| Decision making [1, 4, 7, 9, 13, 15, 16, 18, 19] | 5 [1, 4, 7, 9, 18] | 2 [1, 19] | 7 [1, 4, 7, 9, 13-16, 18, 19] | 2 [4, 9] | 2 [7, 19] | 5 [1, 4, 7, 9, 18] | 2 [7, 9] | 1 [19] | 3 [4, 7, 9] | 1 [4] | 1 [7] | 11 (100) |
| Self-tailoring [4, 6, 9-11, 14, 20, 22] | 4 [4, 6, 9-11] | 1 [22] | 6 [4, 6, 9-11, 20, 22] | 3 [4, 9-11] | 1 [22] | 4 [4, 6, 9-11] | 3 [9, 20, 22] | 3 [6, 10, 11, 20] | 3 [4, 9-11] | 2 [4, 10, 11] | — | 10 (91) |
| Action planning [2, 13-18, 20, 21, 24] | 4 [17, 18, 21, 24] | — | 7 [2, 13-18, 20, 21, 24] | 2 [2, 21] | — | 4 [17, 18, 21, 24] | 1 [20] | 2 [20, 21] | 1 [17] | 1 [21] | — | 8 (73) |
| Maintaining patient-provider partnership [4, 8, 10-12, 20, 24] | 4 [4, 8, 10-12, 24] | — | 5 [4, 8, 10-12, 20, 24] | 2 [4, 10, 11] | — | 4 [4, 8, 10-12, 24] | 1 [20] | 2 [10, 11, 20] | 2 [4, 10, 11] | 2 [4, 10, 11] | — | 8 (73) |
| Resource utilization [7] | 1 [7] | — | 1 [7] | — | 1 [7] | 1 [7] | 1 [7] | — | 1 [7] | — | 1 [7] | 7 (64) |
| Self-management support components |  |  |  |  |  |  |  |  |  |  |  |  |
| Information about condition and/or its management [4, 6-8, 10-15, 17, 18, 21, 23, 24] | 9 [4, 6-8, 10-12, 17, 18, 21, 24] | — | 11 [4, 6-8, 10-18, 21, 23, 24] | 3 [4, 10, 11, 21] | 1 [7] | 9 [4, 6-8, 10-12, 17, 18, 21, 24] | 1 [7] | 3 [6, 10, 11, 21] | 4 [4, 7, 10, 11, 17] | 3 [4, 10, 11, 21] | 1 [7] | 10 (91) |
| Practical support with adherence (medication or behavioral) [4, 7-15, 17-19, 24] | 8 [4, 7-12, 17, 18, 24] | 1 [19] | 10 [4, 7-19, 24] | 3 [4, 9-11] | 2 [7, 19] | 8 [4, 7-12, 17, 18, 24] | 2 [7, 9] | 2 [10, 11, 19] | 5 [4, 7, 9-11, 17] | 2 [4, 10, 11] | 1 [7] | 11 (100) |
| Monitoring of condition with feedback [1, 3, 4, 7, 13-15, 20, 22, 24] | 5 [1, 3, 4, 7, 24] | 3 [1, 3, 22] | 8 [1, 3, 4, 7, 13-16, 20, 22, 24] | 1 [4] | 3 [3, 7, 22] | 5 [1, 3, 4, 7, 24] | 3 [7, 20, 22] | 1 [20] | 2 [4, 7] | 1 [4] | 1 [7] | 11 (100) |
| Training/rehearsal for practical self-management activities [2, 9, 13-18, 20] | 3 [9, 17, 18] | — | 6 [2, 9, 13-18, 20] | 2 [2, 9] | — | 3 [9, 17, 18] | 2 [9, 20] | 1 [20] | 2 [9, 17] | — | — | 7 (64) |
| Provision of easy access to advice or support when needed [4, 8, 10-12, 24] | 4 [4, 8, 10-12, 24] | — | 4 [4, 8, 10-12, 24] | 2 [4, 10, 11] | — | 4 [4, 8, 10-12, 24] | — | 1 [10, 11] | 2 [4, 10, 11] | 2 [4, 10, 11] | — | 7 (64) |
| Social support [4, 8, 12-15, 17, 18, 21] | 5 [4, 8, 12, 17, 18, 21] | — | 6 [4, 8, 12-18, 21] | 2 [4, 21] | — | 5 [4, 8, 12, 17, 18, 21] | — | 1 [21] | 2 [4, 17] | 2 [4, 21] | — | 7 (64) |
| Lifestyle advice and support [18, 21] | 2 [18, 21] | — | 2 [18, 21] | 1 [21] | — | 2 [18, 21] | — | 1 [21] | — | 1 [21] | — | 6 (55) |
| Training/rehearsal for everyday activities [5] | — | 1 [5] | — | — | — | — | — | 1 [5] | — | — | — | 2 (18) |
| Training/rehearsal for psychological strategies [14, 15] | — | — | 1 [13-16] | — | — | — | — | — | — | — | — | 1 (9) |
| Self-management focus areas |  |  |  |  |  |  |  |  |  |  |  |  |
| Pressure ulcer management [1, 3, 4, 7, 10, 11, 13-16, 21, 23] | 6 [1, 3, 4, 7, 10, 11, 21] | 2 [1, 3] | 8 [1, 3, 4, 7, 10, 11, 13-16, 21, 23] | 3 [4, 10, 11, 21] | 2 [3, 7] | 6 [1, 3, 4, 7, 10, 11, 21] | 1 [7] | 2 [10, 11, 21] | 3 [4, 7, 10, 11] | 3 [4, 10, 11, 21] | 1 [7] | 11 (100) |
| Physical activity promotion [6, 13, 16-18, 20, 22] | 3 [6, 17, 18] | 1 [22] | 6 [6, 13-18, 20, 22] | — | 1 [22] | 3 [6, 17, 18] | 2 [20, 22] | 2 [6, 20] | 1 [17] | — | — | 8 (73) |
| Bladder management [4, 8, 12-16, 21, 24] | 4 [4, 8, 12, 21, 24] | — | 5 [4, 8, 12-16, 21, 24] | 2 [4, 21] | — | 4 [4, 8, 12, 21, 24] | — | 1 [21] | 1 [4] | 2 [4, 21] | — | 7 (64) |
| Therapeutic exercise for hands, legs or trunk [2, 5, 9] | 1 [9] | 1 [5] | 2 [2, 9] | 2 [2, 9] | — | 1 [9] | 1 [9] | 1 [5] | 1 [9] | — | — | 8 (73) |
| Psychosocial support [4, 13-15, 17] | 2 [4, 17] | — | 3 [4, 13-17] | 1 [4] | — | 2 [4, 17] | — | — | 2 [4, 17] | 1 [4] | — | 6 (55) |
| Bowel management [13-16, 21] | 1 [21] | — | 2 [13-16, 21] | 1 [21] | — | 1 [21] | — | 1 [21] | — | 1 [21] | — | 6 (55) |
| Pain management [13-16, 24] | 1 [24] | — | 2 [13-16, 24] | — | — | 1 [24] | — | — | — | — | — | 3 (27) |
| Medicating and dieting [13-15, 17] | 1 [17] | — | 2 [13-17] | — | — | 1 [17] | — | — | 1 [17] | — | — | 4 (36) |
| Spasticity management [13, 14] | — | — | 1 [13-16] | — | — | — | — | — | — | — | — | 1 (9) |
| Autonomic dysreflexia management [13] | — | — | 1 [13-16] | — | — | — | — | — | — | — | — | 1 (9) |
| Sleep management [17] | 1 [17] | — | 1 [17] | — | — | 1 [17] | — | — | 1 [17] | — | — | 4 (36) |
| Shoulder posture monitoring [19] | — | 1 [19] | 1 [19] | — | 1 [19] | — | — | 1 [19] | — | — | — | 4 (36) |

^a^Not reported

## References

1. Goodwin, B.M., et al., *Visualization of user interactions with a pressure mapping mobile application for wheelchair users at risk for pressure injuries.* Assistive technology : the official journal of RESNA, 2021: p. 1-Oct.

2. Fizzotti, G., et al., *Tablet Technology for Rehabilitation after Spinal Cord Injury: A Proof-of-Concept.* 2015. 210: p. 479-483.

3. Olney, C.M., et al., *Development of a comprehensive mobile assessment of pressure (CMAP) system for pressure injury prevention for veterans with spinal cord injury.* The journal of spinal cord medicine, 2019. 42(6): p. 685-694.

4. Kryger, M.A., et al., *The Effect of the Interactive Mobile Health and Rehabilitation System on Health and Psychosocial Outcomes in Spinal Cord Injury: Randomized Controlled Trial.* Journal of medical Internet research, 2019. 21(8): p. e14305.

5. Estes, L.T., D. Backus, and T. Starner, *A wearable vibration glove for improving hand sensation in persons with Spinal Cord Injury using Passive Haptic Rehabilitation.* 2015: p. 37-44.

6. Wilroy, J.D., et al., *Correlates of adherence in a home-based, self-managed exercise program tailored to wheelchair users with spinal cord injury.* Spinal cord, 2021. 59(1): p. 55-62.

7. Khan, A. and N. Phung, *Undergraduate research in assistive technology: Design and development of a preventive weight shifting app to reduce the risk of pressure ulcers in wheelchair bound patients with spinal cord injuries (Phase 1).* Journal of Medical Devices, Transactions of the ASME, 2016. 10(2).

8. Wilde, M.H., et al., *Feasibility of a Web-Based Self-management Intervention for Intermittent Urinary Catheter Users With Spinal Cord Injury.* Journal of wound, ostomy, and continence nursing : official publication of The Wound, Ostomy and Continence Nurses Society, 2016. 43(5): p. 529-538.

9. Kongcharoen, J., et al., *Gamification smartphone application for leg physical therapy.* International journal of online and biomedical engineering, 2019. 15(8): p. 31-41.

10. Amann, J., et al., *Co-designing a Self-Management App Prototype to Support People With Spinal Cord Injury in the Prevention of Pressure Injuries: Mixed Methods Study.* JMIR mHealth and uHealth, 2020. 8(7): p. e18018.

11. Amann, J., et al., *Opportunities and Challenges of a Self-Management App to Support People With Spinal Cord Injury in the Prevention of Pressure Injuries: Qualitative Study.* JMIR mHealth and uHealth, 2020. 8(12): p. e22452.

12. Wilde, M.H., et al., *A Web-based self-management intervention for intermittent catheter users.* Urologic nursing, 2015. 35(3): p. 127.

13. Mortenson, W.B., et al., *Improving Self-Management Skills Among People With Spinal Cord Injury: Protocol for a Mixed-Methods Study.* JMIR research protocols, 2018. 7(11): p. e11069.

14. Singh, G., et al., *Patients' Perspectives on the Usability of a Mobile App for Self-Management following Spinal Cord Injury.* Journal of medical systems, 2019. 44(1): p. 26.

15. Mortenson, W.B., et al., *Development of a self-management app for people with spinal cord injury.* Journal of Medical Systems, 2019. 43(6): p. 1-12.

16. MacGillivray, M.K., et al., *Implementing a self-management mobile app for spinal cord injury during inpatient rehabilitation and following community discharge: A feasibility study.* The journal of spinal cord medicine, 2020. 43(5): p. 676-684.

17. Hoevenaars, D., et al., *Mobile App (WHEELS) to Promote a Healthy Lifestyle in Wheelchair Users With Spinal Cord Injury or Lower Limb Amputation: Usability and Feasibility Study.* JMIR formative research, 2021. 5(8): p. e24909.

18. Cole, M., et al., *Website Redesign of a 16-Week Exercise Intervention for People With Spinal Cord Injury by Using Participatory Action Research.* JMIR rehabilitation and assistive technologies, 2019. 6(2): p. e13441.

19. Wang, Q., et al., *Zishi: A smart garment for posture monitoring.* 2016. 7: p. 3792-3795.

20. Bizzarini, E., et al., *A mobile app for home-based exercise in spinal cord injured persons: Proposal and pilot study.* Digital Health, 2022. 8: p. 20552076211070724.

21. Newman, S.D., S.L. Toatley, and M.D. Rodgers, *Translating a spinal cord injury self-management intervention for online and telehealth delivery: A community-engaged research approach.* The journal of spinal cord medicine, 2019. 42(5): p. 595-605.

22. Hiremath, S.V., et al., *Mobile health-based physical activity intervention for individuals with spinal cord injury in the community: A pilot study.* PloS one, 2019. 14(10): p. e0223762.

23. Shirai, T., et al., *The use of a mobile educational tool on pressure injury education for individuals living with spinal cord injury/disease: a qualitative research study.* Disability and rehabilitation, 2020: p. 1-Oct.

24. Potiart, T., K. Harnphadungkit, and P. Phunchongharn, *Development and Effectiveness Testing of “Punsook”: A Smartphone Application for Intermittent Urinary Catheter Users with Spinal Cord Injury.* Siriraj Medical Journal, 2020. 73(2): p. 99-107.
